# Designing a multi-epitope vaccine to control porcine epidemic diarrhea virus infection using immunoinformatics approaches

**DOI:** 10.3389/fmicb.2023.1264612

**Published:** 2023-09-14

**Authors:** Wei Hou, Heqiong Wu, Sibei Wang, Wenting Wang, Bin Wang, Haidong Wang

**Affiliations:** ^1^College of Veterinary Medicine, Shanxi Agricultural University, Jinzhong, China; ^2^Single Molecule Nanometry Laboratory (Sinmolab), Nanjing Agricultural University, Nanjing, China

**Keywords:** PEDV, S protein, vaccine, multi-epitope, immunoinformatics

## Abstract

Porcine epidemic diarrhea virus (PEDV), a continuously evolving pathogen, causes severe diarrhea in piglets with high mortality rates. However, current vaccines cannot provide complete protection against PEDV, so vaccine development is still necessary and urgent. Here, with the help of immunoinformatics approaches, we attempted to design a multi-epitope vaccine named *rPMEV* to prevent and control PEDV infection. The epitopes of *rPMEV* were constructed by 9 cytotoxic T lymphocyte epitopes (CTLs), 11 helper T lymphocyte epitopes (HTLs), 6 linear B cell epitopes (LBEs), and 4 conformational B cell epitopes (CBEs) based on the S proteins from the four representative PEDV G2 strains. To enhance immunogenicity, porcine β-defensin-2 (PBD-2) was adjoined to the N-terminal of the vaccine as an adjuvant. All of the epitopes and PBD-2 were joined by corresponding linkers and recombined into the multivalent vaccine, which is stable, antigenic, and non-allergenic. Furthermore, we adopted molecular docking and molecular dynamics simulation methods to analyze the interaction of *rPMEV* with the Toll-like receptor 4 (TLR4): a stable interaction between them created by 13 hydrogen bonds. In addition, the results of the immune simulation showed that *rPMEV* could stimulate both cellular and humoral immune responses. Finally, to raise the expression efficiency, the sequence of the vaccine protein was cloned into the pET28a (+) vector after the codon optimization. These studies indicate that the designed multi-epitope vaccine has a potential protective effect, providing a theoretical basis for further confirmation of its protective effect against PEDV infection *in vitro* and *in vivo* studies.

## 1. Introduction

Coronaviruses (CoVs) are members of the *Coronaviridae* family with a genome of up to 32 kb that can infect a wide variety of animals and cause enteric, respiratory, and other diseases (Woo et al., 2006). Currently, the most relevant swine enteric coronaviruses include porcine epidemic diarrhea virus (PEDV), transmissible gastroenteritis virus (TGEV), porcine delta coronavirus (PDCoV), and swine acute diarrhea syndrome coronavirus (SADS-CoV) (Wang et al., 2014a; Gong et al., 2017). Among the four known CoVs, PEDV has the highest detection rate and up to 100% mortality in suckling piglets (Zhuang et al., 2022). Since it was first reported in 1971 in the Unite Kingdom (Hou et al., 2021), the same or distinct PEDV strains have spread to many countries, such as the United States, Canada, Peru, and China (Wang et al., 2019). The emergence and reemergence of PEDV have resulted in significant economic losses in the swine industry.

PEDV is an enveloped virus with a single-stranded, positive-sense RNA genome with a size of approximately 28 kb. The genome encodes open reading frame (ORF) 1 ab, spike (S) protein, accessory proteins (ORF3), envelope (E) protein, membrane (M) protein, and nucleocapsid (N) protein from the 5′ to 3′ untranslated region (UTR) (Kocherhans et al., 2001). Among these proteins, the S protein, a type I glycoprotein and receptor-binding protein, is crucial for viral entry and interactions with hosts (Li et al., 2020, 2021). Furthermore, the S protein is also the primary target for stimulating the host cell immune response and inducing neutralizing antibodies (Sun et al., 2008). The S gene is the most changeable section in the PEDV genome, such as in the S gene insertion and deletion strains found in the United States in 2013–2014 (Vlasova et al., 2014; Wang et al., 2014b), thus the S protein bears the greatest evolutionary pressure. Based on phylogenetic analysis of the complete S protein, PEDV was divided into G1 and G2 types. The G1 group consists of three subgroups (G1a, G1b, and G1c) (Hsueh et al., 2020), while the G2 group consists of four subgroups (G2a, G2b, G2c, and G2d) (Li et al., 2022). According to the studies, G2 genotype variants accounted for 92.3% of the major causal viral strains of the PEDV prevalence in China (Li et al., 2022; Zhuang et al., 2022). Currently, due to the high occurrence of PEDV as well as the variety and varying degrees of recombination in its genome, PEDV is still the leading cause of piglet death in Chinese pig farms (Zhuang et al., 2022). Therefore, vaccine development is still essential to preventing and controlling PEDV infection, especially G2 genotype variants.

To prevent and control porcine epidemic diarrhea (PED), many efforts have been undertaken to develop effective vaccinations, such as modified-live vaccines (MLVs) and whole virus-inactivated vaccines (WIVs) (Collin et al., 2015; Gerdts and Zakhartchouk, 2017; Shi et al., 2017; Li et al., 2018). These vaccines have the whole antigenic part of the pathogen and can elicit long-lasting immunity (Moyle and Toth, 2013). However, due to the shedding of MLV and the limited antigen dose of WIV (Collin et al., 2015; Lia et al., 2017; Shi et al., 2017), as well as their production based on restricted strains and the risk of the virulence property, their protection against highly pathogenic or emerging pathogenic strains is limited (Li et al., 2022). Compared with the above vaccine, peptide vaccines, which can contain different specific pathogen proteins and stimulate the body to produce corresponding antibodies, are more suitable for fighting virus variation and solving the problems caused by MLVs and WIVs (Francis, 2018; Yu et al., 2022). In peptide vaccine development, the efficient screening and design of immunogens is a major challenge since short peptides have small molecular weights and usually cause weak immunogenic effects (Sun et al., 2022). Immunoinformatics approaches, which can avoid complicated and difficult manipulation as well as substantial time and financial investment, have become an invaluable tool for epitope localization and epitope discovery (Dong et al., 2020). According to reports, immunoinformatic techniques could increase epitope discovery by 10–20 times while reducing the workload of experiments by 95% (De Groot et al., 2002). Many studies have reported epitope-based peptide vaccines against human and/or animal viruses. For example, Yu et al. (2022) used immunoinformatics tools to design a recombinant multivalent epitope vaccine against SARS-CoV-2 and its variants. Nguyen et al. (2022) developed a novel multi-epitope vaccine-based bioinformatics strategy to prevent the spread of African swine fever. Therefore, epitope-based peptide vaccines designed using immunoinformatics approaches can be a more useful approach for vaccine development.

In this study, we employed immunoinformatic approaches to predict and design a safe and effective multi-epitope candidate vaccine derived from the S protein of prevalent PEDV G2 variants to confer optimistic protection. The findings of this study can serve as a theoretical foundation for the development of a PEDV multi-epitope vaccine.

## 2. Materials and methods

### 2.1. Collection of targeted protein sequence

The entire sequences of the S protein of PEDV G2 genotype strains were obtained from the National Center for Biotechnology Information (NCBI) database. The antigenicity of all S proteins was predicted using the free online software VaxiJen v2.0.^1^ Four representative PEDV strains with the best antigenicity from each G2 subgroup (G2a, G2b, G2c, and G2d) were chosen to identify antigen epitopes and design vaccine.

### 2.2. Sequence alignment and signal peptide prediction of S proteins

The sequence alignment of the S proteins from CH/SCZJ/2018, LW-L, CH/SDLQ/09/2020, and CH/SCST/04/2020 strains was performed using SnapGene tool,^2^ and the mutant amino acid sites were then screened out. To determine the presence of a signal peptide region in the candidate antigen protein, the signal peptide of the S protein was predicted using SignalP-5.0 server^3^ (Almagro Armenteros et al., 2019).

### 2.3. T lymphocyte epitopes prediction

To predict cytotoxic T lymphocyte epitopes (CTLs), the sequences of S proteins were submitted to the Immune Epitope Database (IEDB) server^4^ (Fleri et al., 2017). The 45 common Swine Leukocyte Antigen (SLA) class I molecules were utilized in the prediction by running the “IEDB-recommended” method for each allele. To estimate CTLs, 9-mer epitopes were screened using parameters such as TAP score > 1.0, IC50 < 500 nM, and proteasome score > 1.0. The dominant epitopes, which simultaneously appeared in three SLA-I alleles in each viral strain and had antigenicity values higher than the threshold (0.4) by using VaxiJen v2.0, were compared and incorporated for vaccine formulation. The common dominant epitopes of the four strains were used to create the final vaccine.

To predict helper T lymphocyte epitopes (HTLs), the sequences of S proteins were assessed using the online server NetMHCIIpan 4.0.^5^ Twenty-seven high-frequency human MHC II (HLA-II) alleles were used to predict epitope peptides. The epitopes with a length of 15 amino acid residues were utilized to estimate HTLs. The threshold for peptides with strong bindings was set to its default value. The dominant epitopes, which simultaneously appeared in three HLA-II alleles in each viral strain and had antigenicity values higher than the threshold (0.4) by using VaxiJen v2.0, were compared and incorporated for vaccine formulation. The common dominant epitopes of the four strains were used to create the final vaccine.

### 2.4. Identification of linear B cell epitopes

Linear B cell epitopes (LBEs) were predicted using the IEDB server with the method of Bepipred Linear Epitope Prediction 2.0 at the default threshold of 0.5. Additionally, the predicted epitopes were screened using VaxiJen v2.0. The common epitopes of the strains were used for vaccine construction.

### 2.5. Modeling of S proteins

The structures of S proteins were generated through modeling utilizing the SWISS-MODEL server.^6^ The reliability of the prediction results was evaluated by Global Model Quality Estimate (GMQE) (Yassim et al., 2022). Ramachandran plot and ProSA-web were used to assess the quality of the models (Waterhouse et al., 2018).

### 2.6. Identification of conformational B cell epitopes

To predict conformational B cell epitopes (CBEs), the online software DiscoTope^7^ with a threshold value of −3.7 was used. The common regions of the predicted epitopes from the four strains were taken as the final conformational epitopes and visualized with PyMol.

### 2.7. Construction of multi-epitope vaccine sequence

The final subunit vaccine was designed by sequentially joining the resulting peptide sequences using suitable linkers. Porcine β-defensin-2 (PBD-2) (37 aa) was used as an adjuvant and fused to the N-terminal via the EAAAK linker. The CTLs, HTLs, and B cell epitopes were linked by AAY, GPGPG, and KK, respectively. The TAT sequence (11 aa) was attached to the C-terminal of the vaccine construct. The recombinant PEDV multi-epitope vaccine was named *rPMEV* in the study.

### 2.8. Prediction of the secondary and tertiary structure of *rPMEV*

The secondary structure of *rPMEV* was predicted using SOPMA online analysis software^8^ (Deléage, 2017). The initial tertiary structure was predicted by Robetta server^9^ (Baek et al., 2021). To improve the structure quality and protein stability, the initial vaccine 3D model was refined by GalaxyRefine server.^10^ To validate the refined tertiary structure, a Ramachandran plot that could display favorable areas of backbone dihedra angels against amino acid residues in 3D structure was performed by the SWISS-MODEL workspace (see text footnote 6) (Waterhouse et al., 2018). In addition, ProSA-web was also employed in the final vaccine protein structure validation.

### 2.9. Evaluation of the physicochemical properties, allergenicity, and antigenicity of *rPMEV*

The online tool Protparam^11^ was used to predict the physicochemical properties of *rPMEV*. To ensure the safety of the vaccine, the online software AllerTOP v2.0^12^ was used to predict the allergenicity of *rPMEV*. Meanwhile, the VaxiJen v2.0 server was applied to evaluate the antigenicity of *rPMEV*.

### 2.10. Molecular docking of *rPMEV* with TLR4

To assess the interaction between the vaccine and the immune receptors, molecular docking of *rPMEV* and the TLR4 receptor (PDB ID: 4G8A) was performed by ClusPro server^13^ (Kozakov et al., 2017). Additionally, Ligplot and PyMol software were used to analyze the interaction interface residues.

### 2.11. Molecular dynamics simulation

To evaluate the structural properties and interaction in *rPMEV* and TLR4 receptor complex, molecular dynamics (MD) simulation was performed using GROMACS (Groningen Machine for Chemical Simulations) (He et al., 2021). First, the AMBER99 force field was used to generate the topology for the protein-ligand complex in all of the MD simulations. Then the protein was solvated in a cubic box of TIP3P water (Grifoni et al., 2020), with at least 1.0 nm distancing the protein from the box edge. To balance the charged protein complex, ions were introduced using a genion tool (Shukla et al., 2018). Moreover, in order to prevent steric conflicts and improper geometry, the solvated electroneutral system was also relaxed through energy minimization. Then, the system equilibration was performed with 100 ps of NVT [substance (N), volume (V), and temperature (T)] equilibration and 100 ps of NPT [substance (N), pressure (P), and temperature (T)] equilibration without restraints. Finally, to analyze the trajectory, MD simulations of 100 ns were performed for the complex system.

### 2.12. Immune simulation

To detect the immune response of *rPMEV* to the host, the C-ImmSim Server^14^ was used to perform the immune simulation (Rapin et al., 2010). The simulation step was set at 1050 (Bhatnager et al., 2021). The time steps were 1, 63, and 126 (one time step corresponds to 8 h). The other parameters were used as the default simulation parameters.

### 2.13. Codon optimization and *in silico* cloning

For cloning, codon optimization was performed by the online tool Java Codon Adaptation Tool (JCat)^15^ (Grote et al., 2005). *Escherichia coli* (Strain K12) was chosen to express the vaccine protein. Codon adaptation index (CAI) and the guanine and cytosine (GC) contents were evaluated. For raising the expression efficiency of the final vaccine protein, pET28a (+) was selected as the vector to express the vaccine protein. Before inserting the nucleotide sequence of the vaccine into the vector, the *XhoI* and *EcoRI* restriction enzymes, which are not contained in the vaccine sequence, were inserted at the N- and C-terminals of the sequence, respectively. Finally, the modified nucleotide sequence of the vaccine was cloned by SnapGene tool (see text footnote 2).

## 3. Results

**Figure 1:**
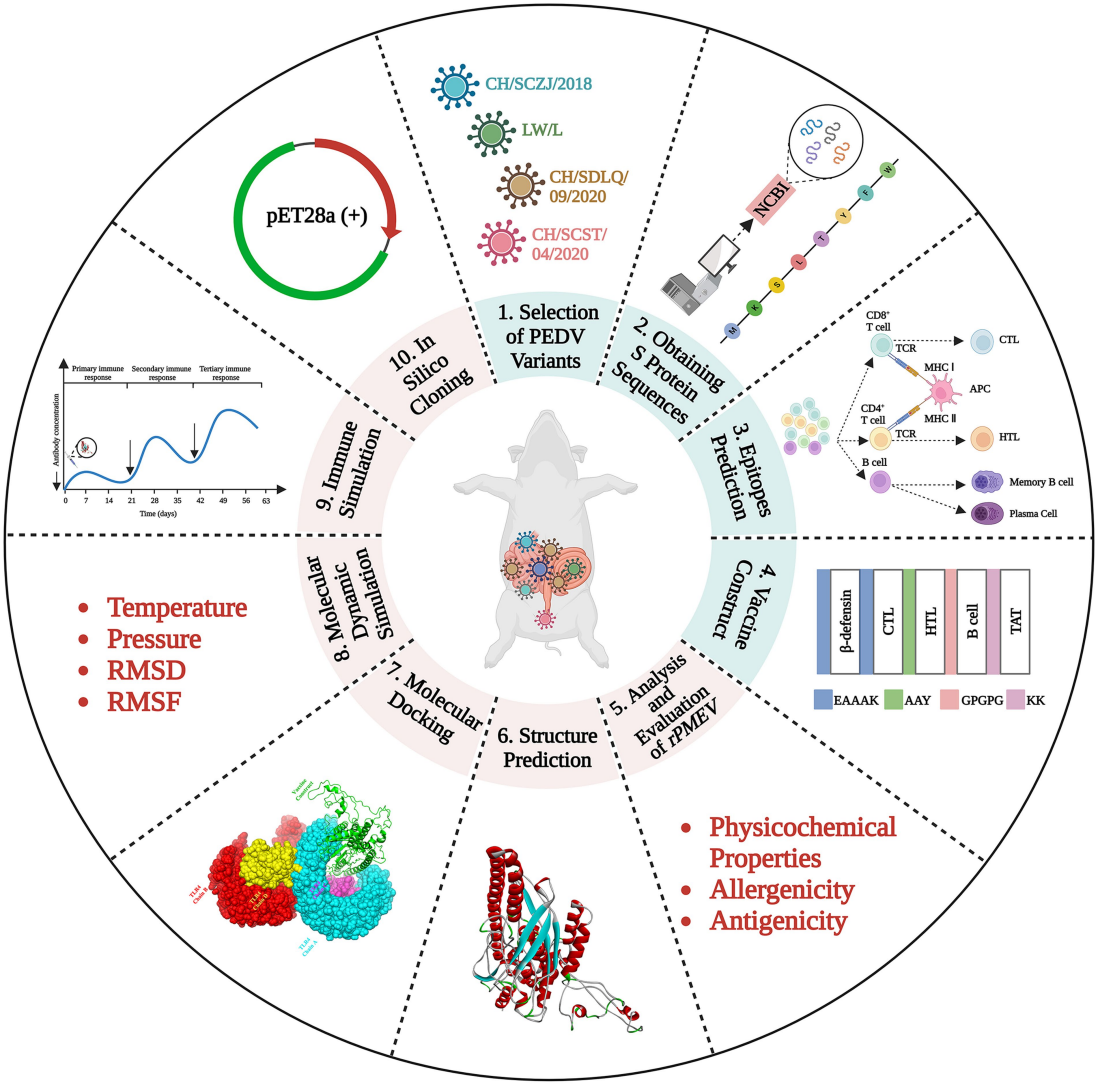
The vaccine construction strategy. The vaccine was designed through many steps, including PEDV variant selection, S protein obtaining, epitope prediction, protein structure prediction, *rPMEV* feature assessment, interaction analysis of *rPMEV* and TLR4, and the evaluation of *rPMEV* immune effect.

The schematic procedure of this research was shown in Figure 1. The vaccine was designed through many steps, including PEDV variant selection, S protein obtaining, epitope prediction, protein structure prediction, *rPMEV* feature assessment, interaction analysis of *rPMEV* and TLR4, and the evaluation of *rPMEV* immune effect. These procedures are mentioned below.

### 3.1. Collection of targeted protein sequence

The GeneBank accession numbers of the four PEDV strains are MH061342.1 (CH/SCZJ/2018), MK392335 (LW/L), MZ161085 (CH/SDLQ/09/2020), and MZ161081 (CH/SCST/04/2020), respectively. The GeneBank accession numbers of S proteins derived from four PEDV strains are AZL47228 (CH/SCZJ/2018), QBM00061 (LW-L), UDL09544 (CH/SDLQ/09/2020), and UDL09540 (CH/SCST/04/2020). The results of the antigenicity of all S proteins are shown in Supplementary Table S1, and the amino acid sequences of these proteins are shown in Supplementary material.

### 3.2. Sequence alignment of S proteins

**Figure 2:**
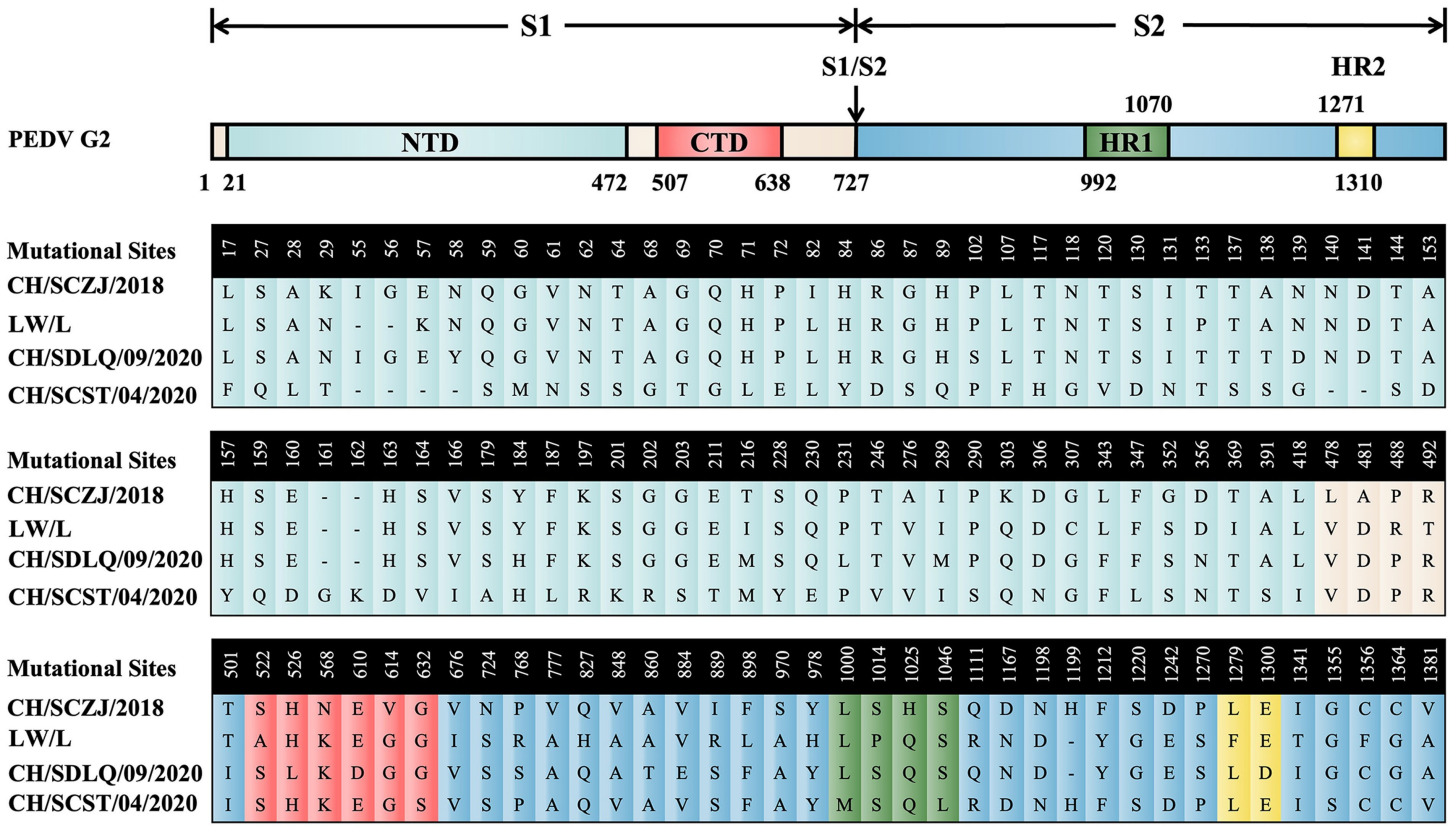
The sequence alignment of PEDV S proteins. The schematic diagram referred to the full-length cDNA structure of PEDV G2 genotype S protein. From the N-terminus to the C-terminus of the S protein, there are 114 unique loci among the four strains, which were located in various domains of the S protein and were displayed in different colors.

To reveal the characteristics of the S proteins from these four PEDV strains, sequence alignment of S proteins was performed. The results, as shown in Figure 2, show that there are 114 distinct loci among the four strains. The S1 region contains 85 sites, 71 of which are in the N-terminal domain (NTD) and 6 in the C-terminal domain (CTD). The S2 region had 29 sites, 4 of which are in heptad repeat region 1 (HR1) and 2 in HR2. The conserved areas of S proteins are the non-mutant areas.

### 3.3. Prediction of signal peptide

After prediction, signal peptide sections were found in S protein of all four PEDV strains, as shown in Supplementary Figure S1. The signal peptide region of CH/SCZJ/2018 is 1–18 (MKSLTYFWLFLPVLSTLS) (Supplementary Figure S1A), the signal peptide sequence of LW/L is (MKSLTYFWLFLPVLSTLS) (Supplementary Figure S1B), the signal peptide sequence of CH/SDLQ/09/2020 is (MKSLTYFWLFLPVLSTLS) (Supplementary Figure S1C), and the signal peptide sequence of CH/SCST/04/2020 is (MKSLTYFWLFLPVLSTFS) (Supplementary Figure S1D). All of the signal peptide sequences were removed for the later epitope prediction of these four PEDV strains.

### 3.4. The identification of CTLs

**Figure 3:**
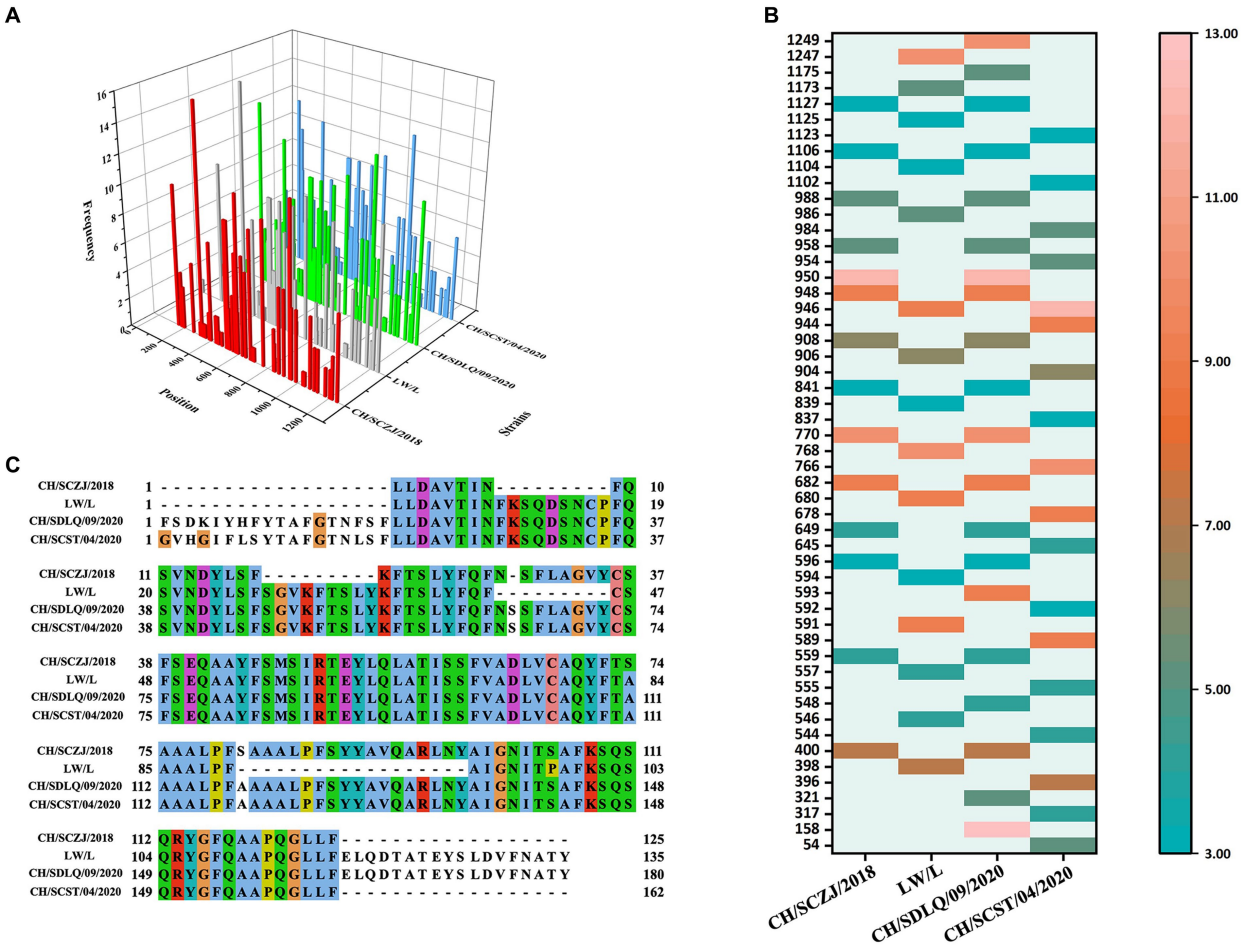
The identification of CTLs. **(A)** After identifying the 45 available SLA-I alleles, all the CTLs of the S protein in the four PEDV strains were shown here. The x-axis indicates the position of the CTLs, the y-axis indicates the strains, and the z-axis indicates the frequency of the epitope in 45 alleles. **(B)** All the dominant CTLs of the S protein in the four PEDV strains were shown here. The abscissa represents the four strains, and the ordinate represents the position of the epitope peptide. The darker colors represent higher frequency. **(C)** All epitopes of each virus strain were connected in sequence according to the size of the starting position, and all common epitopes of all strains were used for vaccine construction through sequence alignment.

The CTLs (9-mers) of 4 PEDV strains were obtained by the IEDB server, and each virus strain has 45 kinds of CTLs of SLA-I alleles. All CTLs were screened with a TAP score > 1.0, IC50 < 500 nM, and proteasome score > 1.0, as shown in Figure 3A. The dominant CTLs for each virus strain that appear in three SLA-I alleles simultaneously and have antigenicity values over the threshold (0.4) were chosen, and they were ranked from small to large according to the sequence start position as shown in Figure 3B. The dominant epitopes of each virus strain are listed in Supplementary Tables S2–S5. The common dominant epitopes of the four strains, as shown in Figure 3C, were compared and integrated for vaccine design.

### 3.5. The identification of HTLs

**Figure 4:**
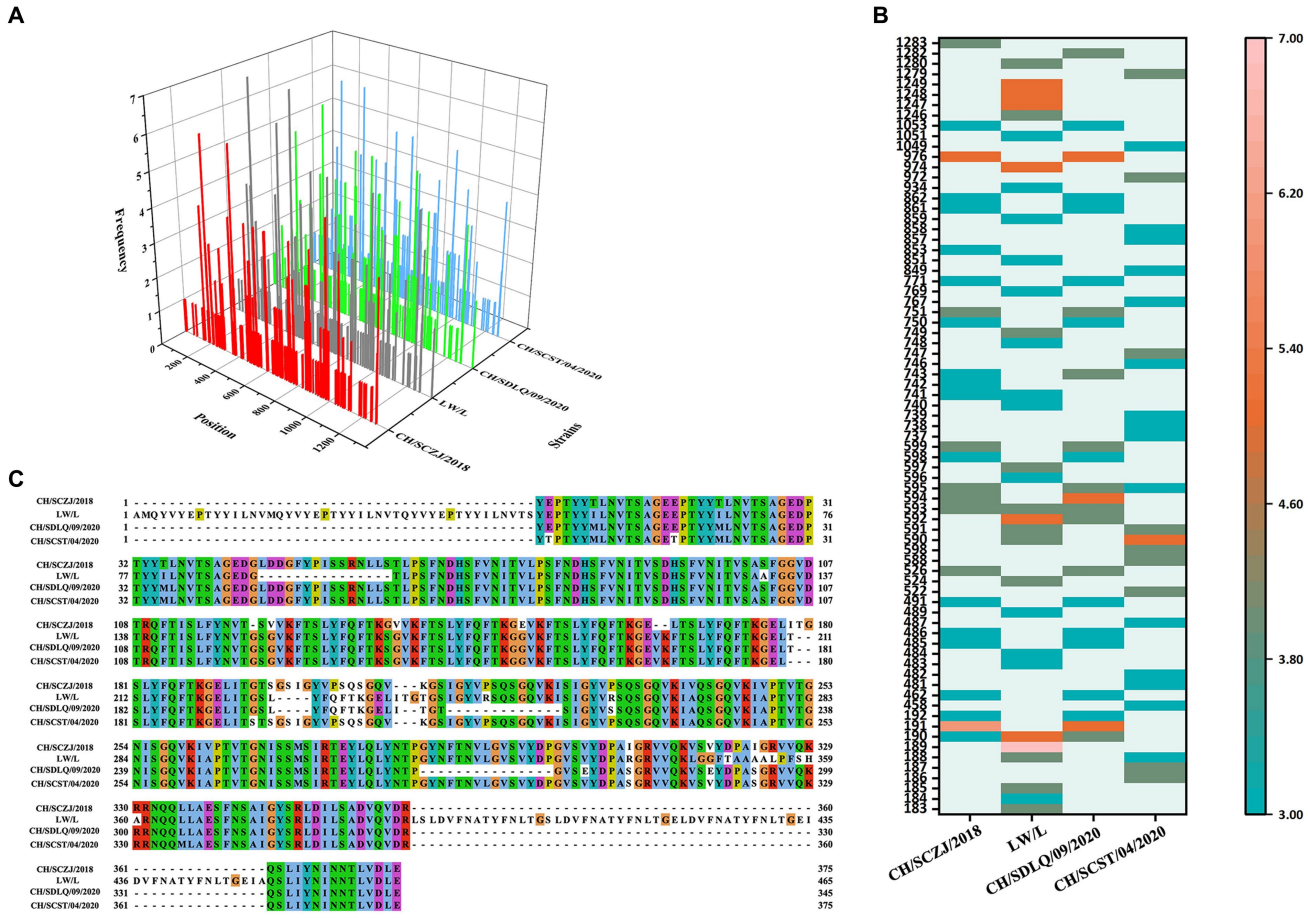
The identification of HTLs. **(A)** After identifying the 27 available HLA-II alleles, all the HTLs of the S protein in the four PEDV strains were shown here. The x-axis indicates the position of the HTLs, the y-axis indicates the strains, and the z-axis indicates the frequency of the epitope in 27 alleles. **(B)** All the dominant HTLs of the S protein in the four PEDV strains were shown here. The abscissa represents the four strains, and the ordinate represents the position of the epitope peptide. The darker colors represent higher frequency. **(C)** All epitopes of each virus strain were connected in sequence according to the size of the starting position, and all common epitopes of all strains were used for vaccine construction through sequence alignment.

The HTLs of four PEDV strains were analyzed by online NetMHCIIpan 4.0 server. The strong binder peptides with a % Rank less than 1.0 were included in this study, as shown in Figure 4A. For each virus strain, the dominant HTLs that appear in three HLA-II alleles at the same time and have antigenicity values greater than the threshold (0.4) were chosen and ranked from small to large based on the sequence start position, as shown in Figure 4B. The dominant epitopes of each virus strain are listed in Supplementary Tables S6–S9. The common dominant epitopes of the four strains, as shown in Figure 4C, were compared and integrated for vaccine design.

### 3.6. The identification of LBEs

**Figure 5:**
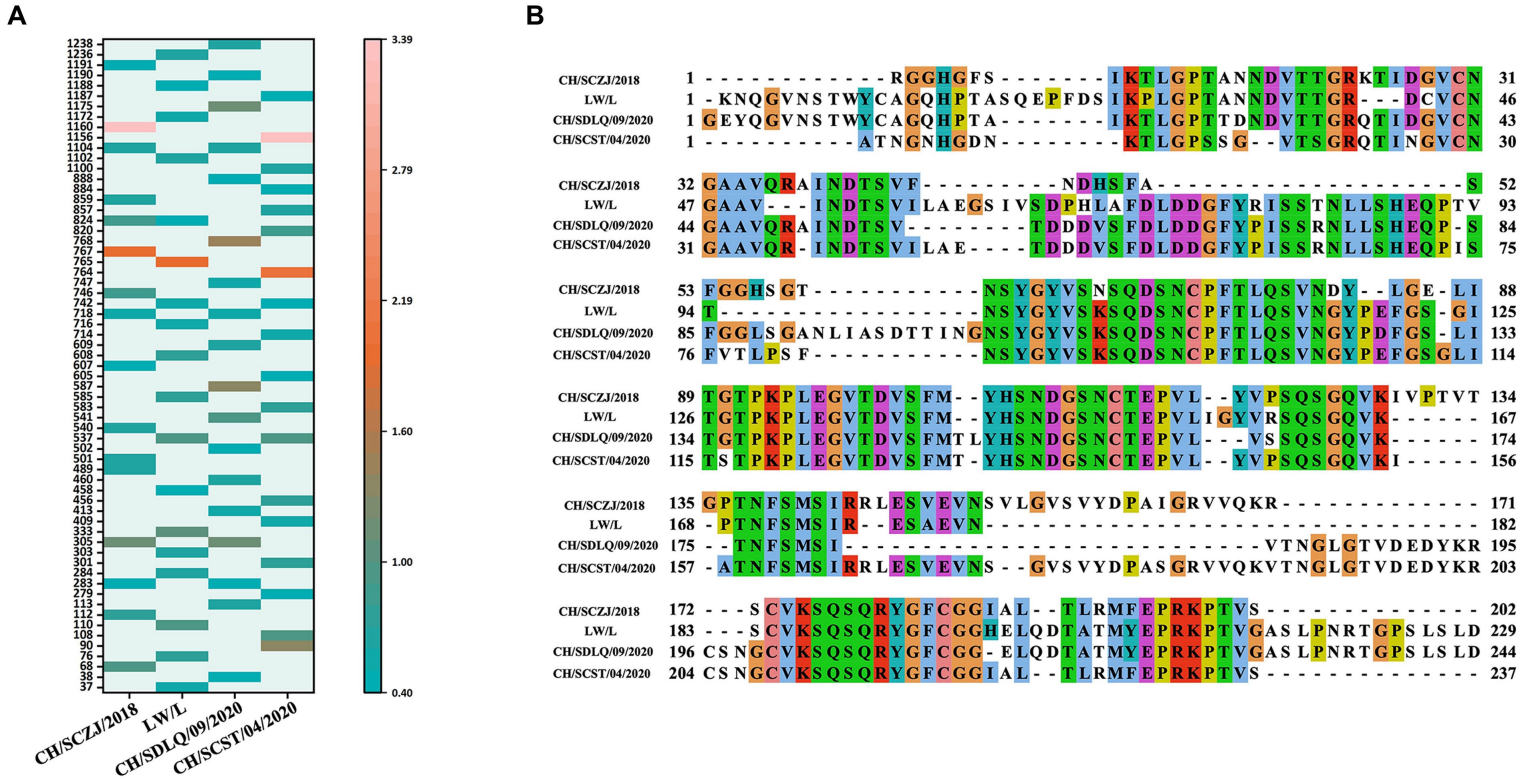
The identification of LBEs. **(A)** All the dominant LBEs of the S protein in the four PEDV strains were shown here. The abscissa represents the four strains, and the ordinate represents the position of the epitope peptide. The darker colors represent higher antigenicity values. **(B)** All epitopes of each virus strain were connected in sequence according to the size of the starting position, and all common epitopes of all strains were used for vaccine construction through sequence alignment.

The LBEs of four PEDV S proteins were predicted by IEDB software. The peptide sequence was identified as an epitope when the peptide threshold in the BepiPred-2.0 method is larger than 0.5 (Supplementary Figure S2). The dominant LBEs were identified based on peptide epitopes with antigenicity values larger than the threshold (0.4), as shown in Figure 5A. The dominant LBEs of four virus strains were compared, as indicated in Figure 5B, and the common dominant LBEs for the four strains were employed to produce the final vaccine (Supplementary Table S10).

### 3.7. Modeling of S proteins

Based on target-template alignment, the 3D structure of S proteins was modeled by SWISS-MODEL server. The GMQE scores of the four S protein models are around 0.70. All models were verified by ProSA-web, which can calculate the Z-Score of models. The Z-Score of CH/SCZJ/2018 model is −10.62, the LW/L model is −10.71, the CH/SDLQ/09/2020 model is −10.81, and the CH/SCST/04/2020 model is −10.71, as shown in Supplementary Figures S3A–D, respectively. The Ramachandran plot drawn by SWISS-MODEL showed that the model residues of CH/SCZJ/2018, LW/L, CH/SDLQ/09/2020, and CH/SCST/04/2020 in the Ramachandran favored region are 94.98%, 95.68%, 95.66%, and 95.79%, respectively, as shown in Supplementary Figures S3E–H. These findings suggested that the S protein models of the four PEDV strains are of high quality and accuracy.

### 3.8. The identification of CBEs

**Figure 6:**
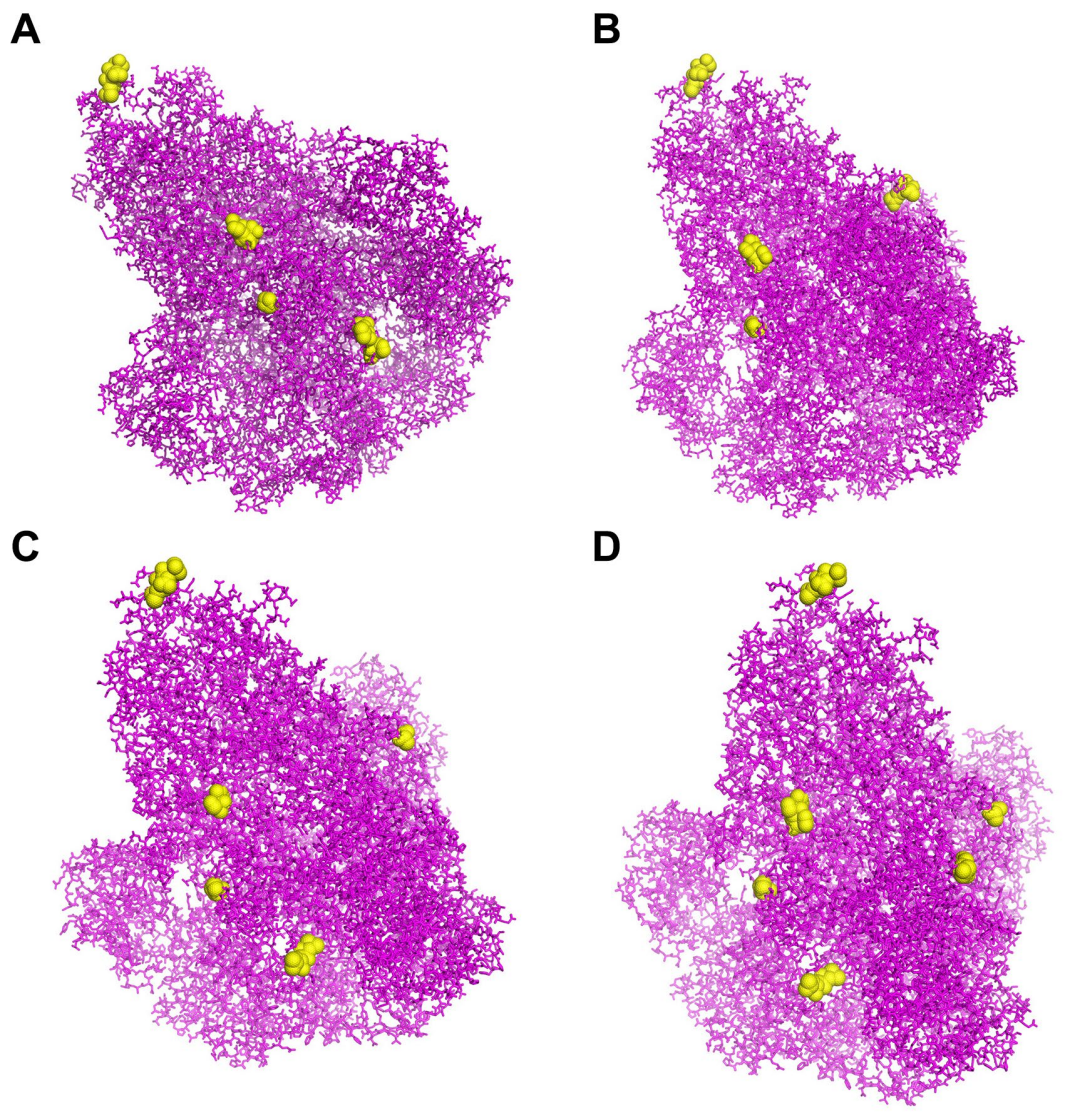
The CBEs of PEDV S protein. **(A)** The “yellow” regions were the selected conformational B cell epitopes of the S protein of virus strain CH/SCZJ/2018. **(B)** The “yellow” regions were the selected conformational B cell epitopes of the S protein of virus strain LW/L. **(C)** The “yellow” regions were the selected conformational B cell epitopes of the S protein of virus strain CH/SDLQ/09/2020. **(D)** The “yellow” regions were the selected conformational B-cell epitopes of the S protein of virus strain CH/SCST/04/2020.

To predict CBEs of each strain, the created models of S proteins were submitted to the online software DiscoTope. All the CBEs of each strain are represented in Supplementary Table S11. The positions of selected conformational epitope residues for each S protein are displayed in Figure 6.

### 3.9. Construction of multi-epitope vaccine sequence

**Figure 7:**
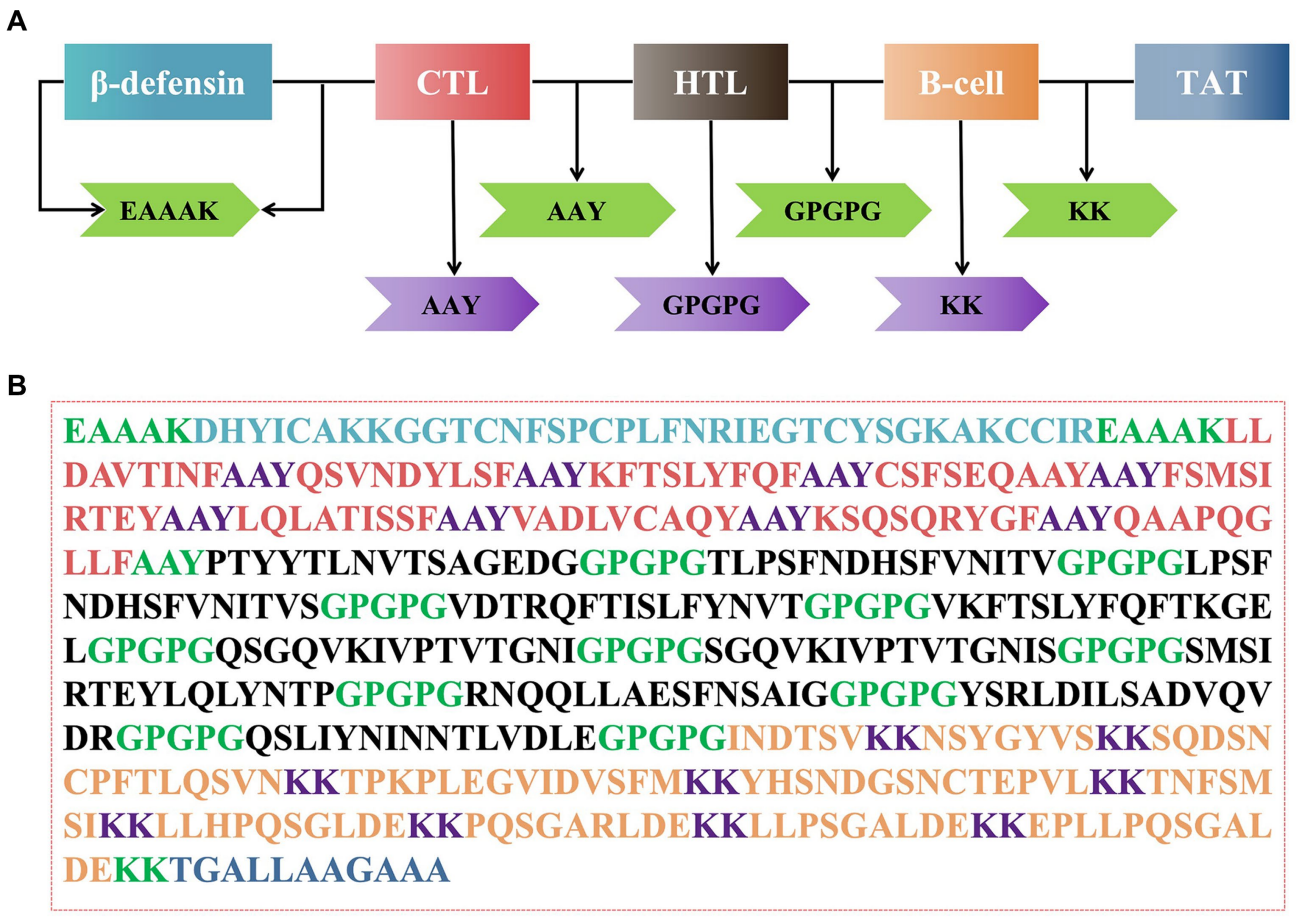
The construction of the multi-epitope vaccine sequence. **(A)** Schematic representation of multi-epitope vaccine construct. **(B)** The amino acid sequence of all the epitopes and linkers required for vaccine construction, and the color of each part corresponded to the color in **(A)**.

The *rPMEV* construct was developed by joining the best candidate antigen epitopes. There are 9 CTLs, 11 HTLs, 6 LBEs, and 4 CBEs in *rPMEV*, as shown in Figure 7A. The CTLs were linked by the AAY linker, which helps the epitopes produce suitable sites for binding to the TAP transporter and enhance epitope presentation (Ayyagari et al., 2022). The HTLs were linked by the GPGPG linker, which can simulate HTL responses and conserve conformation-dependent immunogenicity of helpers as well as antibody epitopes (Ayyagari et al., 2022). The LBEs and CBEs were joined using the KK linker, which can increase immunogenicity (Li et al., 2015). Furthermore, at the N-terminus, porcine β-defensin-2 (PBD-2) (37 aa) was added to the vaccine construct to increase the immunogenicity of the multi-epitope vaccine using the EAAK linker, which can effectively separate proteins while reducing their contact (Kumar Pandey et al., 2018). To enable intercellular delivery, a TAT sequence was appended to the vaccine construct at the C-terminal. The number of vaccine residues in the final designed multivalent vaccine is 508 aa, as seen in Figure 7B.

### 3.10. The prediction of *rPMEV* secondary structure

The secondary structure of *rPMEV* was analyzed using SOPMA server. The results show that there is 27.56% alpha helix, 23.03% extended strand, 5.51% beta turn, and 43.90% random coil, as illustrated in Supplementary Figure S4.

### 3.11. The prediction, refinement, and validation of *rPMEV* tertiary structure

Based on the sequence, the Robetta server was used to model the tertiary structure of *rPMEV*, and a total of five models were output. The quality and potential errors in the five models were verified by ProSA-web, as shown in Supplementary Figure S5. The Z-Score indicates overall model quality. The Z-Scores of models 1–5 are −7.16, −7.2, −5.4, −6.69, and −5.52, respectively. Model 1 was adopted as the initial model of *rPMEV* (Supplementary Figure S6A) since it has a lower Z-Score and is therefore of higher quality (Supplementary Figure S6B). The Ramachandran plot drawn by SWISS-MODEL workspace reveals that the initial model has 93.68% Ramachandran favored region, 1.58% Ramachandran outlier region, and 0.00% Rotamer outlier region, as shown in Supplementary Figure S6C. To optimize the tertiary of the vaccine, the initial model was refined by GalaxyRefine Server, and there were five optimized models output by the server. Similar to the initial model selection, all the optimized models were further verified by ProSA-web as shown in Supplementary Figure S7. The Z-Scores of the optimized models 1–5 are −7.39, −7.25, −7.29, −7.36, and −7.31, respectively. The optimized Model 1 was chosen as the final tertiary structure of *rPMEV* (Supplementary Figure S6D) since it has the lowest Z-Score and is of the highest quality (Supplementary Figure S6E). The Ramachandran plot drawn by SWISS-MODEL workspace shows that the final tertiary structure is 95.06% of residues in the Ramachandran favored region, 0.40% in the Ramachandran outlier region, and only 0.99% in the Rotamer region, as shown in Supplementary Figure S6F.

### 3.12. Evaluation of *rPMEV* physicochemical properties

The vaccine protein is composed of 508 amino acids and 7,506 atoms, with the molecular formula C_2412_H_3714_N_630_O_736_S_14_. The molecular weight of *rPMEV* is 54 KD, indicating that it is a suitable vaccine because proteins with molecular weights less than 110 KD can be efficiently purified (Barh et al., 2013). The theoretical pI of *rPMEV* is 8.59, and it includes 37 negatively charged residues (Asp and Glu) and 43 positively charged residues (Arg and Lys). The instability index (II) of *rPMEV* is 34.74, and the vaccine is classified as a stable protein since it is smaller than the instability index value of 40 (a value above 40 predicts that the protein is unstable) (Zhao et al., 2019). The aliphatic index of *rPMEV* is 71.50, which is calculated as the proportional volume occupied by aliphatic side chains. The grand average of hydropathicity (GRAVY) is −0.244. Since the GRAVY ranges from −2 to 2, negative values indicate that the protein is hydrophilic (Sha et al., 2020), so *rPMEV* belongs to a hydrophilic nature.

### 3.13. Analysis of *rPMEV* allergenicity and antigenicity

To avoid autoimmune reactions, an allergenicity analysis of *rPMEV* was performed using AllerTOP v2.0. Both *rPMEV* and the nearest protein, UniProtKB accession number O1449, are defined as non-allergens. The antigenicity of *rPMEV* predicted by VaxiJen v2.0 is 0.654, which is more than the threshold of 0.4. Therefore, allergenicity and antigenicity studies suggest that *rPMEV* is a safe protective antigen.

### 3.14. Molecular docking of *rPMEV* with TLR4

**Figure 8:**
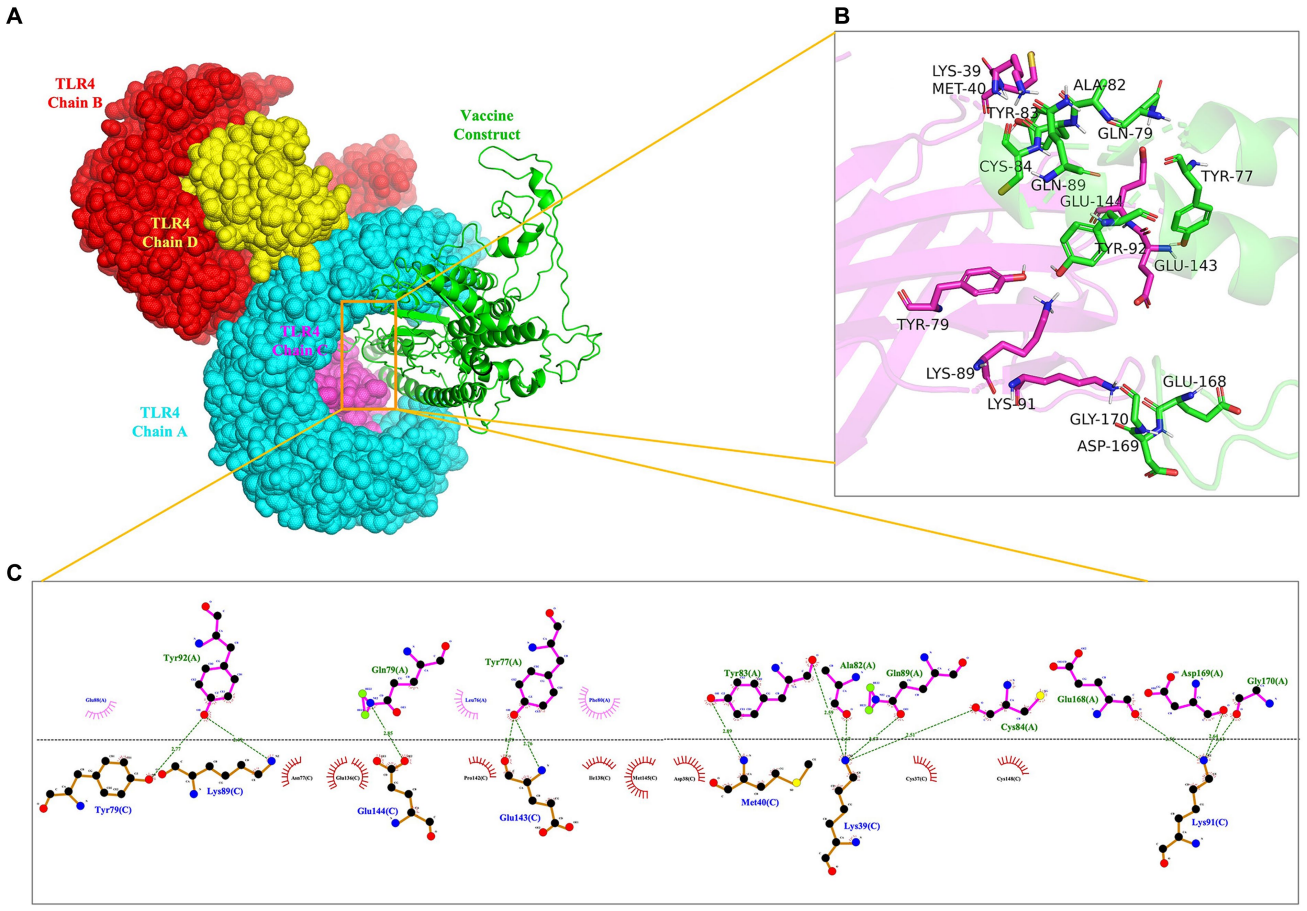
The molecular docking of *rPMEV* with TLR4. **(A)** Docked complex molecule of TLR4 in four different colors (chain A in cyan, chain B in red, chain C in magenta, and chain D in yellow) and vaccine construct in green. **(B)** The interaction interface predicted by PyMol in 3D. **(C)** The interaction interface predicted by Ligplot in 2D. The green dotted line represents the hydrogen bond.

To evaluate the interaction affinity between *rPMEV* and TLR4, molecular docking was carried out using ClusPro Server. The interaction of *rPMEV* with TLR4 produces 30 *rPMEV*-TLR4 complexes, and all of the docking data are displayed in Supplementary Table S12. The conformation with the least interaction energy (−1046.1 kcal/mol) obtained by the docking of the *rPMEV*-TLR4 complex is shown in Figure 8A. The interaction interface residues were analyzed by PyMol in 3D and Ligplot in 2D format, as seen in Figures 8B,C, respectively. The results demonstrate that there are 13 hydrogen bonds at the docking interface engaged in the interaction of the complex subunits.

### 3.15. Molecular dynamics simulation

**Figure 9:**
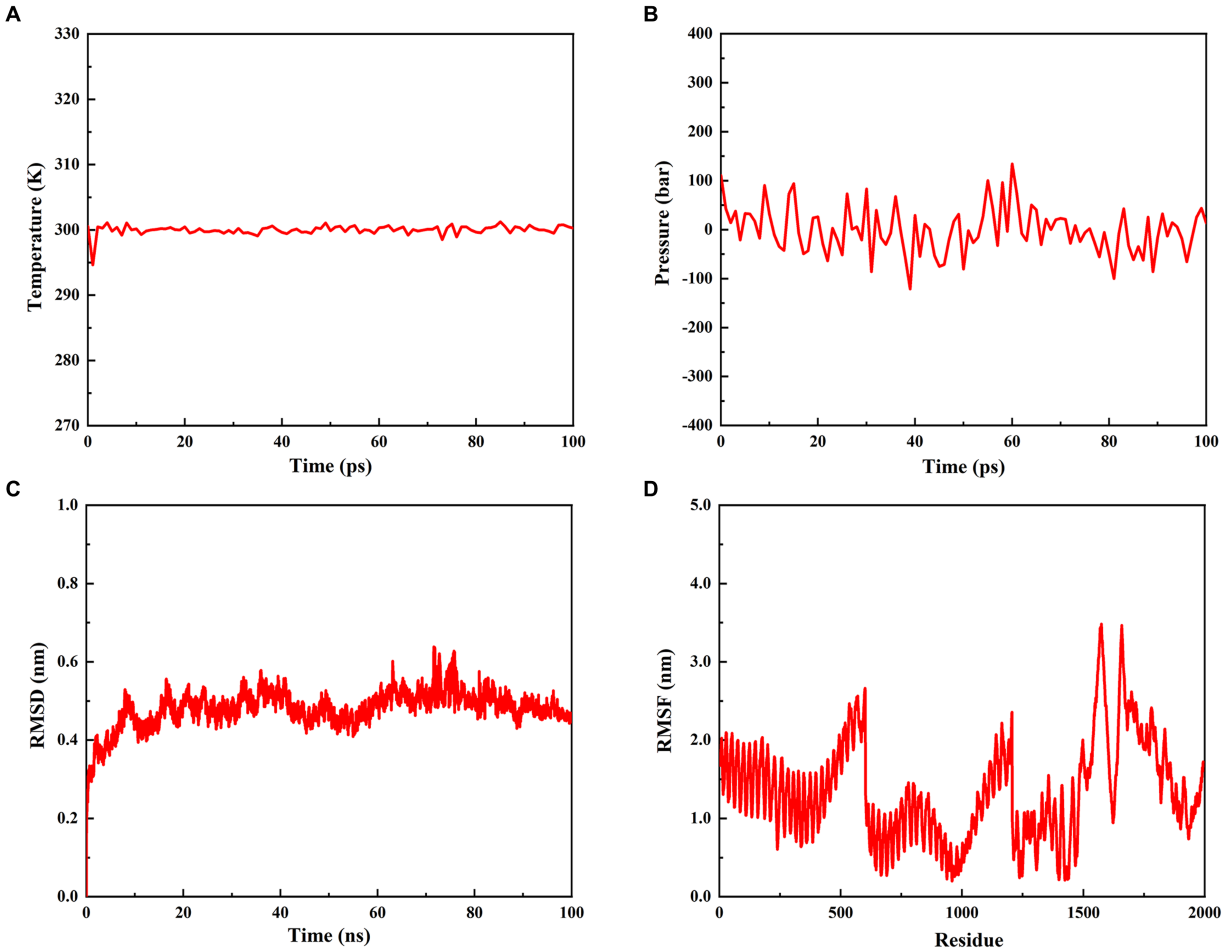
Molecular dynamics simulation of the *rPMEV*-TLR4 complex. **(A)** The temperature plot of the *rPMEV*-TLR4 complex (constant at 300 K for 100 ps). **(B)** The pressure plot of the *rPMEV*-TLR4 complex (displaying fluctuation at 1.7 bar value for 100 ps). **(C)** The RMSD analysis of the *rPMEV*-TLR4 complex. **(D)** The RMSF analysis of the *rPMEV*-TLR4 complex.

To estimate the stability of the *rPMEV*-TLR4 complex, MD simulation was performed using GROMACS. During the MD simulation, energy minimization, temperature, pressure, RMSD, and RMSF were analyzed. As shown in Figures 9A,B, the temperature and pressure plots indicate that the system is around 300 K and 1.7 atmospheres with 100 ps of the time interval, indicating that the system is stable and the MD operation is successful. The RMSD against time of the *rPMEV*-TLR4 complex, which represents the structural fluctuation of the overall structure of the protein complex, reveals a large fluctuation during the 0–8 ns simulation. After 8 ns, the RMSD value is kept around 0.5 nm, indicating the stability of the vaccine complex (Figure 9C). In addition, the RMSF of the *rPMEV*-TLR4 complex, which indicates the flexibility of the residue in the docking complex, was also performed. The majority of amino acid residues in the *rPMEV*-TLR4 complex are below 2.0 nm, and only a few residues have larger changes, indicating that the complex has stability and stiffness (Figure 9D).

### 3.16. Immune simulation

**Figure 10:**
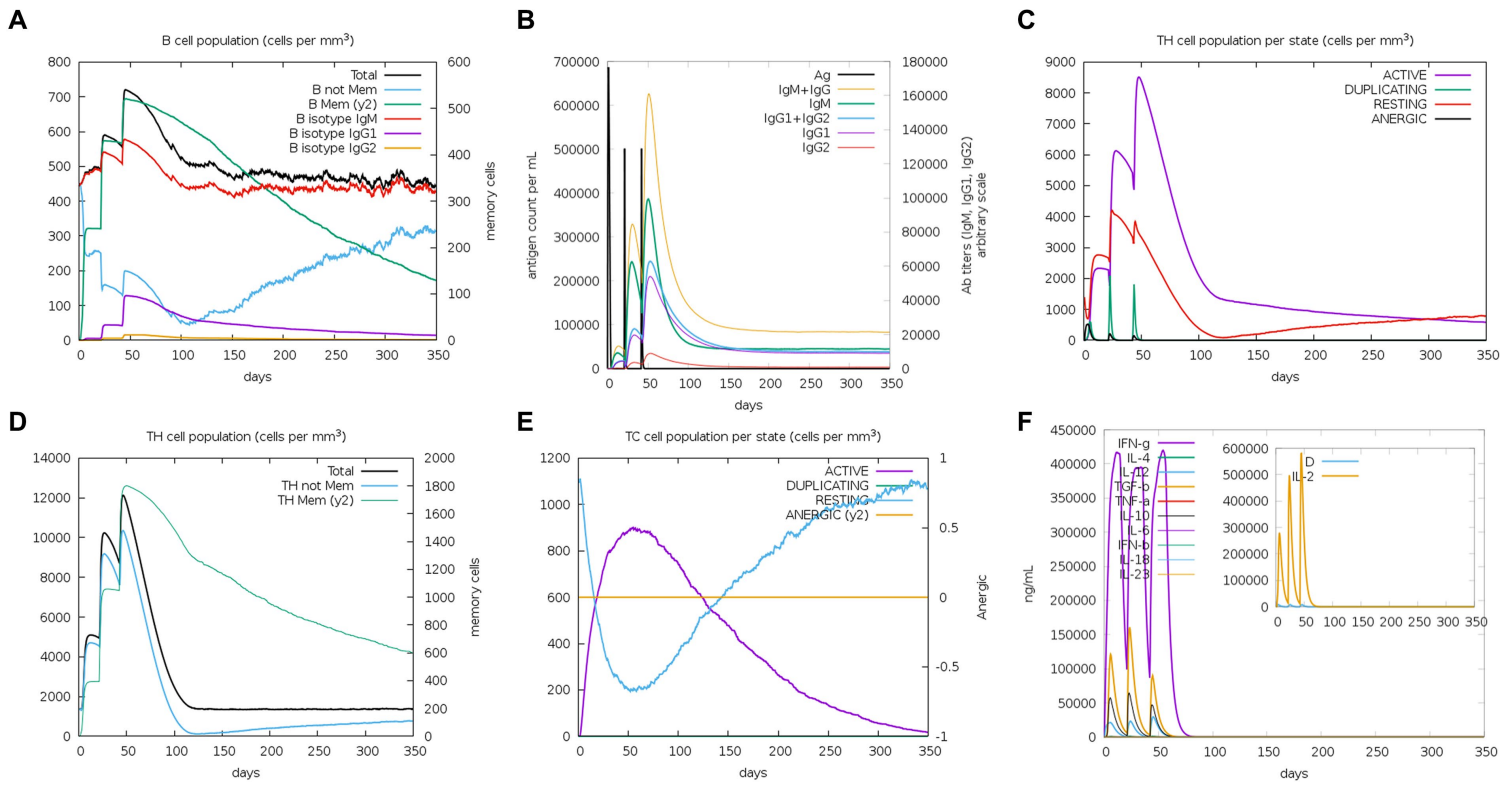
C-ImmSim simulation profile of PEDV final vaccine. **(A)** The B cell population of various subtypes after vaccination. **(B)** The production of immunoglobulin production after vaccination and the response of specific subclass (colored peaks) to injected antigen (black peaks). **(C)** The HTL populations in various states. **(D)** The population of HTL memory cells and not memory cells after vaccination. **(E)** The CTL populations in various states. **(F)** The concentration of cytokines and interleukins against the vaccine antigen.

The C-ImmSim server was used to study the immune stimulation of the final vaccine. The simulation results reveal that the immune response after immunization is mainly composed of B cell (Figure 10A) and T cell populations. After the three immune responses, significant antibody titers are produced against the antigen (Figure 10B). Additionally, after the secondary and tertiary immune responses, the active TH cell populations and the populations of total and memory TH cells are also increased, as shown in Figures 10C,D, respectively. Furthermore, as illustrated in Figure 10E, the population of cytotoxic T cells also increases steadily after each immunization. Importantly, after the three stages of immunization, IFN-g (interferon-gamma) shows a robust response among the cytokines and interleukins (Figure 10F).

### 3.17. Codon optimization and *in silico* cloning

To produce a suitable plasmid construct containing the vaccine construct sequence, codon optimization was undertaken (Supplementary Figure S8). The results of codon optimization of the vaccine construct show that the improved vaccine has a CAI value of 0.947 and a GC-content of 52.56%, indicating that the final vaccine has a high potential for good expression in *E. coli* since CAI >0.8 is regarded as a good value for high-level expression, and the optimal GC content ranges from 30% to 70% (Nezafat et al., 2016). For cloning the vaccine construct into the *E. coli* pET28a (+) vector, restriction sites *XhoI* and *EcoRI* were added to the N- and C- terminals of the codon sequence, and then the sequence of 1,524 bases was inserted into the pET28a (+) vector using SnapGene software, as shown in Supplementary Figure S9.

## 4. Discussion

PEDV is a global pathogen of pigs, with a high incidence in pigs of all ages and a mortality rate of nearly 100% in suckling piglets, and it has caused serious economic losses to the swine industry (Li et al., 2020). According to the latest study, G2 genotype variants accounted for 92.3% of the major causal viral strains of PEDV prevalence in China in the most recent years (2020–2021) (Zhuang et al., 2022). And the current vaccines cannot provide complete protection against PEDV (Su et al., 2022). Therefore, effective vaccines that can prevent and control PEDV are an urgent need for pig industries worldwide (Li et al., 2022). Based on the ability of vaccine protein elicited mainly by PEDV S protein, we contrived a multivalent epitope vaccine of S protein in this study to effectively solve the G2 genotype variants problem of PEDV epidemic.

As getting the relative antigen epitopes is essential for designing an epitope vaccination (Li et al., 2013), the T cell epitopes of S protein of four G2 strains (CH/SCZJ/2018 of G2a, LW/L of G2b, CH/SDLQ/09/2020 of G2c, and CH/SCST/04/2020 of G2d) were first screened by a variety of epitope prediction software, which can improve the accuracy of epitope prediction. T cell epitopes include two types. One is the CTL epitope, which is crucial to defending against viral infections by recognizing intracellular viral pathogens via MHC class I molecules (Dong et al., 2020). The other is the HTL epitope, which plays an important role in the antiviral immune response by producing IFN-γ and also inducing and maintaining CTL response (Dong et al., 2020). In order to expand the broader protective effect of the vaccine, 45 available SLA class I molecules and 27 high-frequency HLA-II alleles were used to predict the T cell epitope peptides of PEDV, which is consistent with the method used to predict multiple epitope vaccination for African swine fever (Ros-Lucas et al., 2020). Finally, 9 CTLs and 11 HTLs were predicted for each of the four strains.

B cell epitopes, in addition to T cell epitopes, are essential components of vaccine design because they play a significant role in humoral immunity by generating antibodies and memory cells to protect against future contacts with the same pathogens (Kumar Pandey et al., 2018). Linear epitopes and conformational epitopes are two types of B cell epitopes that are widely predicted in epitope vaccine design. For example, Ding et al. used immunoinformatics approaches to predict a recombinant vaccine containing 9 linear B cell epitopes and 4 conformational B cell epitopes for the prevention and control of COVID-19 (Yu et al., 2022). Chauhan et al. (2019) designed a multi-epitope vaccine against Kaposi Sarcoma using immunoinformatics approaches, which had 9 linear B cell epitopes and 7 conformational B cell epitopes. Recently, Polyiam et al. (2022) predicted the B cell epitopes of PEDV S protein using immunoinformatics methods. In their study, however, no conformational B cell epitopes were predicted. To predict a comprehensive PEDV multi-epitope vaccine, both linear and conformational B cell epitopes based on the S protein of four PEDV strains were screened in our study. Finally, we screened out 6 linear B cell epitopes and 4 conformational B cell epitopes to construct the vaccine.

To conjugate and efficiently separate the epitopes to facilitate the process of antigen presentation (Chen et al., 2013), peptide linkers were positioned between each epitope. In *rPMEV*, there are 9 CTLs, 11 HTLs, 6 LBEs, and 4 CBEs. The CTLs were connected by AAY, the HTLs by GPGPG, and the LBEs and CBEs were connected by KK. In addition, it has been reported that PBD-2 is highly expressed in porcine epithelial cells and possesses antibacterial activity, immunoregulation, and intestinal tract protection (Han et al., 2015). Recombinant PBD-2 has been used to prevent postweaning diarrhea in piglets and enhance their growth performance when added to their feed (Peng et al., 2016). Therefore, combined with the above availability and advantages of PBD-2, we incorporated PBD-2 (37 aa) into the vaccine construct with the help of the EAAAK linker. Moreover, a TAT sequence was appended at the C-terminal of the vaccine construct because TAT sequences can carry macromolecules, allowing them to penetrate cell membranes more easily, thus promoting the phagocytosis of vaccine proteins by APCs (Frankel and Pabo, 1988). Finally, the multivalent vaccine contains 614 amino acids.

After the design and construction of the vaccine, the secondary structure was analyzed. The results show that the vaccine protein has 5.51% beta turn and 43.90% random coil, indicating that the vaccine has a good structural basis since the loose spatial arrangement of beta turn and random coil facilitates the formation of epitopes (Yu et al., 2022). The tertiary structure of the vaccine was predicted using the Robetta server and then further refined using the GalaxyRefine server. Additionally, the physicochemical properties, allergenicity, and antigenicity of the designed vaccine constructs were then evaluated, and the results from these analyses demonstrate that the r*PMEV* is reasonable, hydrophilic, and safe.

In addition, it has been reported that the binding of vaccine structural molecules with host immune receptors is necessary to activate a cellular and humoral immune response against the target pathogen (Alharbi et al., 2022). TLR4 is a transmembrane protein that belongs to the Toll-like receptor (TLR) family. Its activation can result in the intracellular signaling pathway of NF-kB and cytokine release, which leads to innate immune system activation and eventually results in durable adaptive immunity (Vaure and Liu, 2014). Moreover, it was reported that PBD-2 could directly interact with TLR4 by isothermal titration calorimetry and far-Western blot *in vitro* (Huang et al., 2019). Thus, molecular docking between *rPMEV* and TLR4 was performed by the ClusPro server to understand the binding pattern between them. The docking interface proved that the lowest energy-weighted score of the docking complex is −1046.1 cal/mol, which is mainly stabilized by a network of 13 hydrogen bond interactions. In addition, the MD simulations performed by GROMACS further suggested the stability of the interface interaction between *rPMEV* and TLR4 through its calculated parameters like temperature, pressure, and RMSD, suggesting the ability of *rPMEV* to induce TLR pathways.

Importantly, similar to other studies (Ismail et al., 2022; Yu et al., 2022), we also used the C-ImmSim server to predict the *in vitro* immunogenicity of *rPMEV*. The results showed that immune response was generally increased after the three stages of immunization, such as increased levels of cytokines IFN-γ and IL-12, which play roles in viral replication inhibition, T-cell and natural killer cell production, and viral clearance (Jiang et al., 2023), indicating that the vaccine structure is expected to induce cellular and humoral immune responses.

Furthermore, to ensure the expression of the designed vaccine in a specific expression system, the codon was optimized. The optimized sequence of the designed vaccine was modified by introducing the *XhoI* and *EcoRI* restriction sites at the N- and C-terminal positions, respectively. Subsequently, the optimized sequence was successfully incorporated into the pET28a (+) vector with these two restriction sites. But the safety and efficacy of the designed vaccine for PEDV should be further studied.

In many current studies on the design of viral vaccines based on viral proteins, there are few studies on designing for the epidemic PEDV strains at the same time. In this study, we have designed a recombinant multivalent epitope vaccine based on the S proteins of PEDV G2 strains with the help of immunoinformatics tools. Starting from considering the epidemic PEDV strains currently, to the restriction of SLA alleles, and then to the interaction between vaccine protein and TLR4 molecule. This work has been innovatively and logically designed in the aforementioned aspects to enhance the immunogenicity and application of PEDV vaccine. Furthermore, we also performed a prediction assessment of the immunogenicity of vaccinations by an online server at the same time. With less time and money spent on trial studies in wet labs, this vaccine design study will help vaccine developers create vaccines more quickly. However, the research findings will finally be enhanced by wet lab validations using model organisms.

## Data availability statement

The original contributions presented in the study are included in the article/Supplementary material, further inquiries can be directed to the corresponding author.

## Author contributions

WH: Writing – original draft, Writing – review & editing. HeW: Writing – review & editing. SW: Writing – review & editing. WW: Writing – review & editing. BW: Writing – review & editing. HaW: Writing – original draft, Writing – review & editing.

## Funding

The author(s) declare financial support was received for the research, authorship, and/or publication of this article. This study was supported by Fundamental Research Program of Shanxi Province (No. 202103021223168), the Shanxi Province Excellent Doctoral Work Award-Scientific Research Project (No. SXBYKY2022053), the Start-up Fund for Doctoral Research, Shanxi Agricultural University (No. 2021BQ71), Shanxi Provincial Key Research and Development Program (No. 202102140601020), the “Six New” Project of Agriculture and Rural Department of Shanxi Province, the Fund for Shanxi “1331 Project” Key Innovative Research Team (No. 20211331–16), the Fund for Shanxi “1331 Project” (No. 20211331–13).

## Conflict of interest

The authors declare that the research was conducted in the absence of any commercial or financial relationships that could be construed as a potential conflict of interest.

## Publisher’s note

All claims expressed in this article are solely those of the authors and do not necessarily represent those of their affiliated organizations, or those of the publisher, the editors and the reviewers. Any product that may be evaluated in this article, or claim that may be made by its manufacturer, is not guaranteed or endorsed by the publisher.

## Supplementary material

The Supplementary material for this article can be found online at: https://www.frontiersin.org/articles/10.3389/fmicb.2023.1264612/full#supplementary-material

## Footnotes

^1^http://www.ddg-pharmfac.net/vaxijen/VaxiJen/VaxiJen.html

^2^http://www.snapgene.com/

^3^http://www.cbs.dtu.dk/services/SignalP/

^4^https://www.iedb.org

^5^https://services.healthtech.dtu.dk/service.php?NetMHCIIpan-4.0

^6^https://swissmodel.expasy.org/

^7^http://tools.iedb.org/discotope/

^8^npsa-prabi.ibcp.fr/cgi-bin/npsa_automat.pl?page=npsa_sopma.html

^9^https://robetta.bakerlab.org

^10^http://galaxy.seoklab.org/

^11^https://web.expasy.org/protparam/

^12^http://www.ddg-pharmfac.net/AllerTOP

^13^https://cluspro.bu.edu/login.php?redir/queue.php

^14^http://kraken.iac.rm.cnr.it/C-IMMSIM/

^15^http://www.jcat.de/Start.jsp
