# Supplementary material for: Designing a multi-epitope vaccine to control porcine epidemic diarrhea virus infection using immunoinformatics approaches
Source: Front Microbiol. 2023 Sep 14;14:1264612. doi: 10.3389/fmicb.2023.1264612 (PMC10538973; doi:10.3389/fmicb.2023.1264612)
Supplement: Supplementary file 1 [file Data_Sheet_1.pdf]

Supplementary Material

1 Supplementary Data

1.1 The amino acid sequence of S proteins

AZL47228:MKSLTYFWLFLPVLSTLSLPQDVTRCSAKTNFRRFFSKFNVQAPAVVVLGGYLPI
GENQGVNSTWYCAGQHPTASGVHGIFISHIRGGHGFEIGISQEPFDPSGYQLYLHKATNGNT
NATARLRICQFPSIKTLGPTANNDVTTGRNCLFNKAIPAHMSEHSVVGITWDNDRVTVFSDKI
YYFYFKNDWSRVATKCYNSGGCAMQYVVYEPTYYTLNVTSAGEDGISYQPCTANCIGYAAN
VFATEPNGHIPEGFSFNNWFLLSNDSTLVHGKVASNQPLLVNCLLAIPKIYGLGQFFSFNKTID
GVCNGAAVQRAPEALRFNINDTSVILAEGSIVLHTALGTNFSFVCGNSSDPHLATFAIPLGAT
QVPYYCFLKVDTYNSTVYKFLAVLPPTVREIVITKYGDVYVNGFGYLHLGLLDAVTINFTGH
GTDDDVSGFWTIASTNFVDALIEVQGTAIQRILYCDDPVSQLKCSQLAFALDDGFYPISSRNL
LSHEQPTSFVTLPSFNDHSFVNITVSASFGGHSGANLIASDTTINGFSSFCVDTRQFTISLFYNV
TNSYGYVSNSQDSNCPFTLQSVNDYLSFSKFCVSTSLLASACTIDLFGYPEFGSVVKFTSLYF
QFTKGELITGTPKPLEGVTDVSFMTLDVCTKYTIYGFKGEGIITLTNSSFLAGVYYTSDSGQLL
AFKNVTSGAVYSVTPCSFSEQAAYVDDDIVGVISSLSNSTFNSTRELPGFFYHSNDGSNCTEP
VLVYSNIGVCKSGSIGYVPSQSGQVKIVPTVTGNISIPTNFSMSIRTEYLQLYNTPVSVDCATY
VCNGNSRCKQLLTQYTAACKTIESALQLSARLESVEVNSMLTISEEALQLATISSFNGDGYNF
TNVLGVSVYDPAIGRVVQKRSFIEDLLFNKVVTNGLGTVDEDYKRCSNGRSVADLVCAQYY
SGVMVLPGVVDAEKLHMYSASLIGGMVLGGFTSAAALPFSYAVQARLNYLALQTDVLQRN
QQLLAESFNSAIGNITSAFESVKEAISHTSKGLNTVAHALTKVQEVVNSQGAALTQLTVQLQ
HNFQAISSSIDDIYSRLDILSADVQVDRLITGRLSALNAFVAQTLTKYTEVQASRKLAQQKVN
ECVKSQSQRYGFCGGDGEHIFSLVQAAPQGLLFLHTVLVPGDFVDVIAIAGLCVNDEIALTLR
EPGLVLFTHELQNHTATEYFVSSRRMFEPRKPTVSDFVQIESCVVTYVNLTRDQLPDVIPDYI
DVNKTLDEILASLPNRTGPSLPLDVFNATYLNLTGEIADLEQRSESLRNTTEELQSLIYNINNT
LVDLEWLNRVETYIKWPWWVWLIIFIVLIFVVSLLVFCCISTGCCGCCGCCCACFSGCCRGPR
LQPYEVFEKVHVQ

QBM00061:MKSLTYFWLFLPVLSTLSLPQDVTRCSANTNFRRFFSKFNVQAPAVVVLGGYLP
KNQGVNSTWYCAGQHPTASGVHGIFLSHIRGGHGFEIGISQEPFDPSGYQLYLHKATNGNTN
ATARLRICQFPSIKPLGPTANNDVTTGRNCLFNKAIPAHMSEHSVVGITWDNDRVTVFSDKIY
YFYFKNDWSRVATKCYNSGGCAMQYVVYEPTYYILNVTSAGEDGISYQPCTANCIGYAANVF
ATEPNGHIPEGFSFNNWFLLSNDSTLVHGKVVSNQPLLVNCLLAIPKIYGLGQFFSFNQTIDC
VCNGAAVQRAPEALRFNINDTSVILAEGSIVLHTALGTNFSFVCSNSSDPHLATFAIPLGAIQV
PYYCFLKVDTYNSTVYKFLAVLPPTVREIVITKYGDVYVNGFGYLHLGLLDAVTINFTGHGT
DDDVSGFWTIASTNFVDALIEVQGTAIQRILYCDDPVSQLKCSQVAFDLDDGFYRISSTNLLS
HEQPTSFVTLPSFNDHSFVNITVSAAFGGHSGANLIASDTTINGFSSFCVDTRQFTISLFYNVT
NSYGYVSKSQDSNCPFTLQSVNDYLSFSKFCVSTSLLASACTIDLFGYPEFGSGVKFTSLYFQ
FTKGELITGTPKPLEGVTDVSFMTLDVCTKYTIYGFKGEGIITLTNSSFLAGIYYTSDSGQLLA
FKNVTSGAVYSVTPCSFSEQAAYVDDDIVGVISSLSSSTFNSTRELPGFFYHSNDGSNCTEPVL
VYSNIGVCKSGSIGYVRSQSGQVKIAPTVTGNISIPTNFSMSIRTEYLQLYNTPVSVDCATYVC
NGNSRCKQLLTHYTAACKTIESALQLSARLESAEVNSMLTISEEALQLATISSFNGDGYNFTN
VLGVSVYDPARGRVVQKRSLIEDLLFNKVVTNGLGTVDEDYKRCSNGRSVADLVCAQYYS

GVMVLPGVVDAEKLHMYSASLIGGMVLGGFTAAAALPFSHAVQARLNYLALQTDVLQRN
QQLLAESFNSAIGNITPAFESVKEAISQTSKGLNTVAHALTKVQEVVNSQGAALTQLTVQLQ
HNFQAISSSIDDIYSRLDILSADVQVDRLITGRLSALNAFVAQTLTKYTEVRASRKLAQQKVN
ECVKSQSQRYGFCGGDGEHIFSLVQAAPQGLLFLHTVLVPGDFVNVIAIAGLCVNDEIALTLR
EPGLVLFTHELQDTATEYFVSSRRMYEPRKPTVGDFVQIESCVVTYVNLTRDQLPEVIPDYID
VNKTLDEILASLPNRTGPSLSLDVFNATYFNLTGEIADLEQRSESLRNTTEELQSLIYNINNTL
VDLEWLNRVETYIKWPWWVWLIIFIVLTFVVSLLVFCCISTGFCGCCGCCGACFSGCCRGPR
LQPYEAFEKVHVQ

UDL09544:MKSLTYFWLFLPVLSTLSLPQDVTRCSANTNFRRFFSKFNVQAPAVVVLGGYLPI
GEYQGVNSTWYCAGQHPTASGVHGIFLSHIRGGHGFEIGISQEPFDSSGYQLYLHKATNGNT
NATARLRICQFPSIKTLGPTTDNDVTTGRNCLFNKAIPAHMSEHSVVGITWDNDRVTVFSDKI
YHFYFKNDWSRVATKCYNSGGCAMQYVYEPTYYMLNVTSAGEDGISYQLCTANCIGYAA
NVFATEPNGHIPEGFSFNNWFLLSNDSTLVHGKVVSNQPLLVNCLLAMPKIYGLGQFFSFNQ
TIDGVCNGAAVQRAPEALRFNINDTSVILAEGSIVLHTAFGTNFSFVCSNSSNPHLATFAIPLG
ATQVPYYCFLKVDTYNSTVYKFLAVLPPTVREIVITKYGDVYVNGFGYLHLGLLDAVTINFT
GHGTDDDVSGFWTIASTNFVDALIEVQGTAIQRILYCDDPVSQLKCSQVAFDLDDGFYPISSR
NLLSHEQPISFVTLPSFNDHSFVNITVSASFGGLSGANLIASDTTINGFSSFCVDTRQFTISLFYN
VTNSYGYVSKSQDSNCPFTLQSVNDYLSFSKFCVSTSLLASACTIDLFGYPDFGSGVKFTSLY
FQFTKGELITGTPKPLEGVTDVSFMTLDVCTKYTIYGFKGEGIITLTNSSFLAGVYYTSDSGQL
LAFKNVTSGAVYSVTPCSFSEQAAYVDDDIVGVISSLSSSTFNSTRELPGFFYHSNDGSNCTEP
VLVYSNIGVCKSGSIGYVSSQSGQVKIAPTVTGNISIPTNFSMSIRTEYLQLYNTPVSVDCATY
VCNGNSRCKQLLTQYTAACKTIESALQLSARLESAEVNSMLTISEETLQLATISSFNGDGYNF
TNVLGVSEYDPASGRVVQKRSFIEDLLFNKVVTNGLGTVDEDYKRCSNGRSVADLVCAQY
YSGVMVLPGVVDAEKLHMYSASLIGGMVLGGFTAAAALPFSYAVQARLNYLALQTDVLQR
NQQLLAESFNSAIGNITSAFESVKEAISQTSKGLNTVAHALTKVQEVVNSQGAALTQLTVQL
QHNFQAISSSIDDIYSRLDILSADVQVDRLITGRLSALNAFVAQTLTKYTEVQASRKLAQQKV
NECVKSQSQRYGFCGGDGEHIFSLVQAAPQGLLFLHTVLVPGDFVNVIAIAGLCVNDEIALT
LREPGLVLFTHELQDTATEYFVSSRRMYEPRKPTVGDFVQIESCVVTYVNLTRDQLPEVIPDY
IDVNKTLDEILASLPNRTGPSLSLDVFNATYLNLTGEIADLEQRSESLRNTTDELQSLIYNINN
TLVDLEWLNRVETYIKWPWWVWLIIFIVLIFVVSLLVFCCISTGCCGCCGCCGACFSGCCRGP
RLQPYEAFEKVHVQ

UDL09540:MKSLTYFWLFLPVLSTFSLPQDVTRCQLTTNFRRFFSKFNVQAPAVVVLGGYLPS
MNSSSWYCGTGLETASGVHGIFLSYIDSGQGFEIGISQEPFDPSGYQFYLHKATNGNHGAVA
RLRICQFPDNKTLGPSSGVTSGRNCLFNKDIPAYMQDGKDVVIGITWDNDRVTVFADKIYHF
YLKNDWSRVATRCYNKRSCAMQYVYTPTYYMLNVTSAGEDGIYYEPCTANCIGYAANVFA
VEPNGHIPEGFSFNNWFLLSNDSTLVHGKVVSNQPLLVNCLLAISKIYGLGQFFSFNQTINGV
CNGAAVQRAPEALRFNINDTSVILAEGSIVLHTAFGTNLSFVCSNSSNPHLATFAIPLGATQVP
YYCFLKVDTYNSTVYKFLSVLPPTVREIVITKYGDVYVNGFGYLHIGLLDAVTINFTGHGTD
DDVSGFWTIASTNFVDALIEVQGTAIQRILYCDDPVSQLKCSQVAFDLDDGFYPISSRNLLSH
EQPISFVTLPSFNDHSFVNITVSASFGGHSGANLIASDTTINGFSSFCVDTRQFTISLFYNVTNS
YGYVSKSQDSNCPFTLQSVNDYLSFSKFCVSTSLLASACTIDLFGYPEFGSGVKFTSLYFQFT
KGELITSTPKPLEGVTDVSFMTLDVCTKYTIYGFKGEGIITLTNSSFLAGVYYTSDSGQLLAFK
NVTSGAVYSVTPCSFSEQAAYVDDDIVGVISSLSSSTFNSTRELPGFFYHSNDGSNCTEPVLV
YSNIGVCKSGSIGYVPSQSGQVKIAPTVTGNISIPTNFSMSIRTEYLQLYNTPVSVDCATYVCN
GNSRCKQLLTQYTAACKTIESALQLSARLESVEVNSMLTISEEALQLATISSFNGDGYNFTNV

LGVSVYDPASGRVVQKRSFIEDLLFNKVVTNGLGTVDEDYKRCSNGRSVADLVCAQYYSG
VMVLPGVVDAEKLHMYSASLIGGMVLGGFTAAAALPFSYAVQARLNYLALQTDVLQRNQ
QMLAESFNSAIGNITSAFESVKEAISQTSKGLNTVAHALTKVQEVVNLQGAALTQLTVQLQH
NFQAISSSIDDIYSRLDILSADVQVDRLITGRLSALNAFVAQTLTKYTEVRASRKLAQQKVNE
CVKSQSQRYGFCGGDGEHIFSLVQAAPQGLLFLHTVLVPGDFVDVIAIAGLCVNDEIALTLRE
PGLVLFTHELQNHTATEYFVSSRRMFEPRKPTVSDFVQIESCVVTYVNLTRDQLPDVIPDYID
VNKTLDEILASLPNRTGPSLPLDVFNATYLNLTGEIADLEQRSESLRNTTEELQSLIYNINNTL
VDLEWLNRVETYIKWPWWVWLIIFIVLIFVVSLLVFCCISTSCCGCCGCCCACFSGCCRGPRL
QPYEVFEKVHVQ

2 Supplementary Figures and Tables

2.1 Supplementary Figures

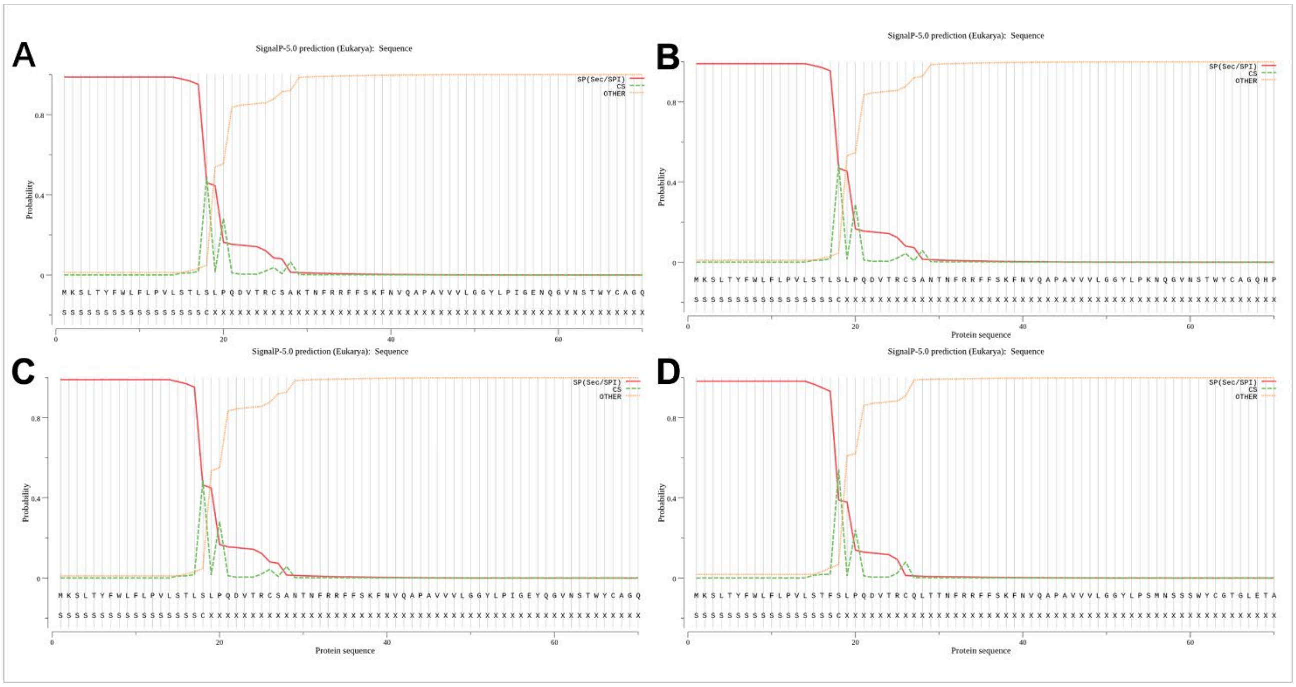

Figure S1. The signal peptides of S proteins. (A) The signal peptide prediction of CH/SCZJ/2018. The probability of signal peptide is 98.74%. **(B)** The signal peptide prediction of LW/L. The probability of signal peptide is 99.01%. **(C)** The signal peptide prediction of CH/SDLQ/09/2020. The probability of signal peptide is 98.95%. **(D)** The signal peptide prediction of CH/SCST/04/2020. The probability of signal peptide is 98.16%. SP (Sec/SPI): type of signal peptide predicted; CS: the cleavage site; Other: the probability that the sequence does not have any kind of signal peptide.

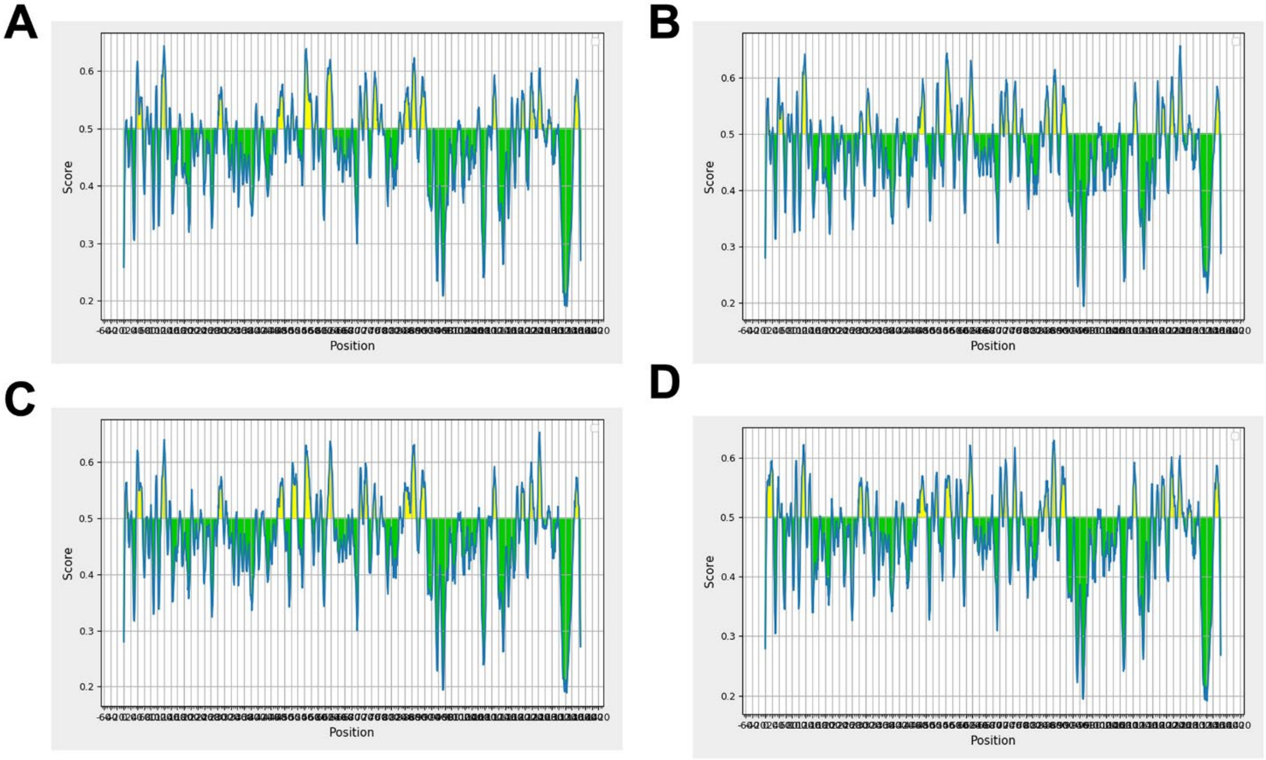

Figure S2. Graphical representation of linear B cell epitopes within the PEDV S protein. (A) The peptide sequence analysis of CH/SCZJ/2018. **(B)** The peptide sequence analysis of LW/L. **(C)** The peptide sequence analysis of CH/SDLQ/09/2020. **(D)** The peptide sequence analysis of CH/SCST/04/2020. The yellow-colored peaks represent the epitopic region, while the green-colored slopes represent the non-epitopic region.

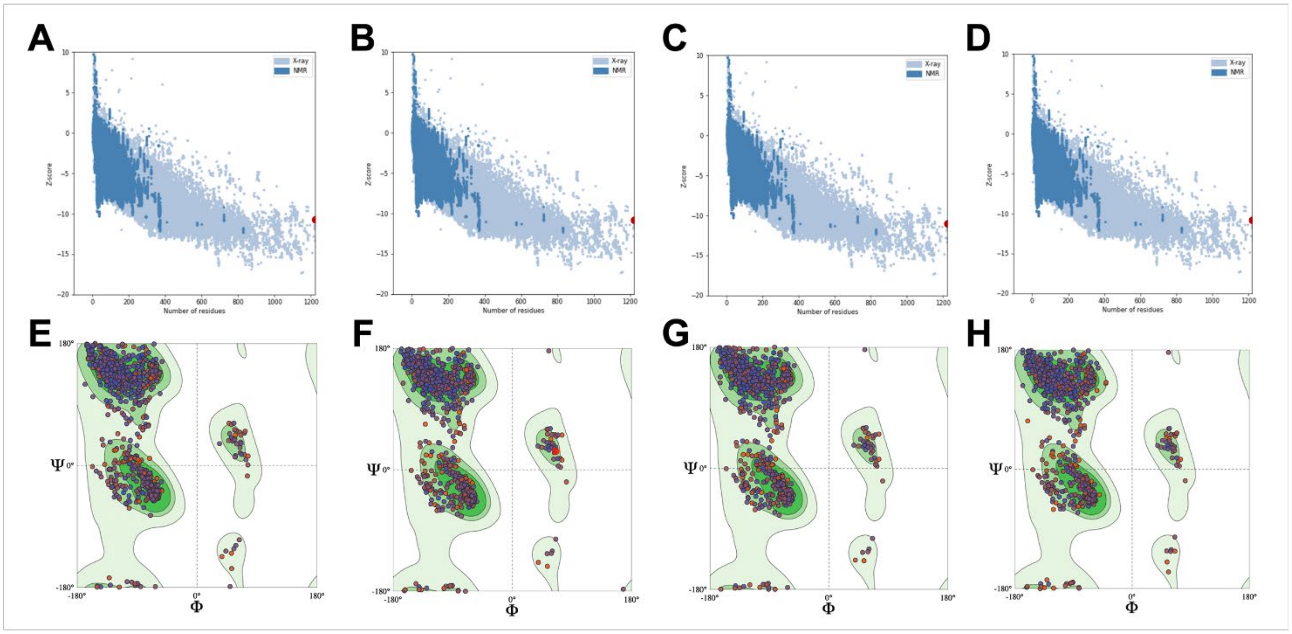

Figure S3. Modeling of S proteins. (A)-(D) Z-Score plots for 3D structure of the S proteins of virus strain CH/SCZJ/2018, LW/L, CH/SDLQ/09/2020, and CH/SCST/04/2020, respectively. Z-Score plot contains Z-Scores of all experimental protein chains in PDB determined by NMR spectroscopy (dark blue) and X-ray crystallography (light blue). The red dot indicates the position of the protein. **(E)-(H)** Ramachandran plots for 3D structure of the S proteins of virus strain CH/SCZJ/2018, LW/L, CH/SDLQ/09/2020, and CH/SCST/04/2020, respectively.

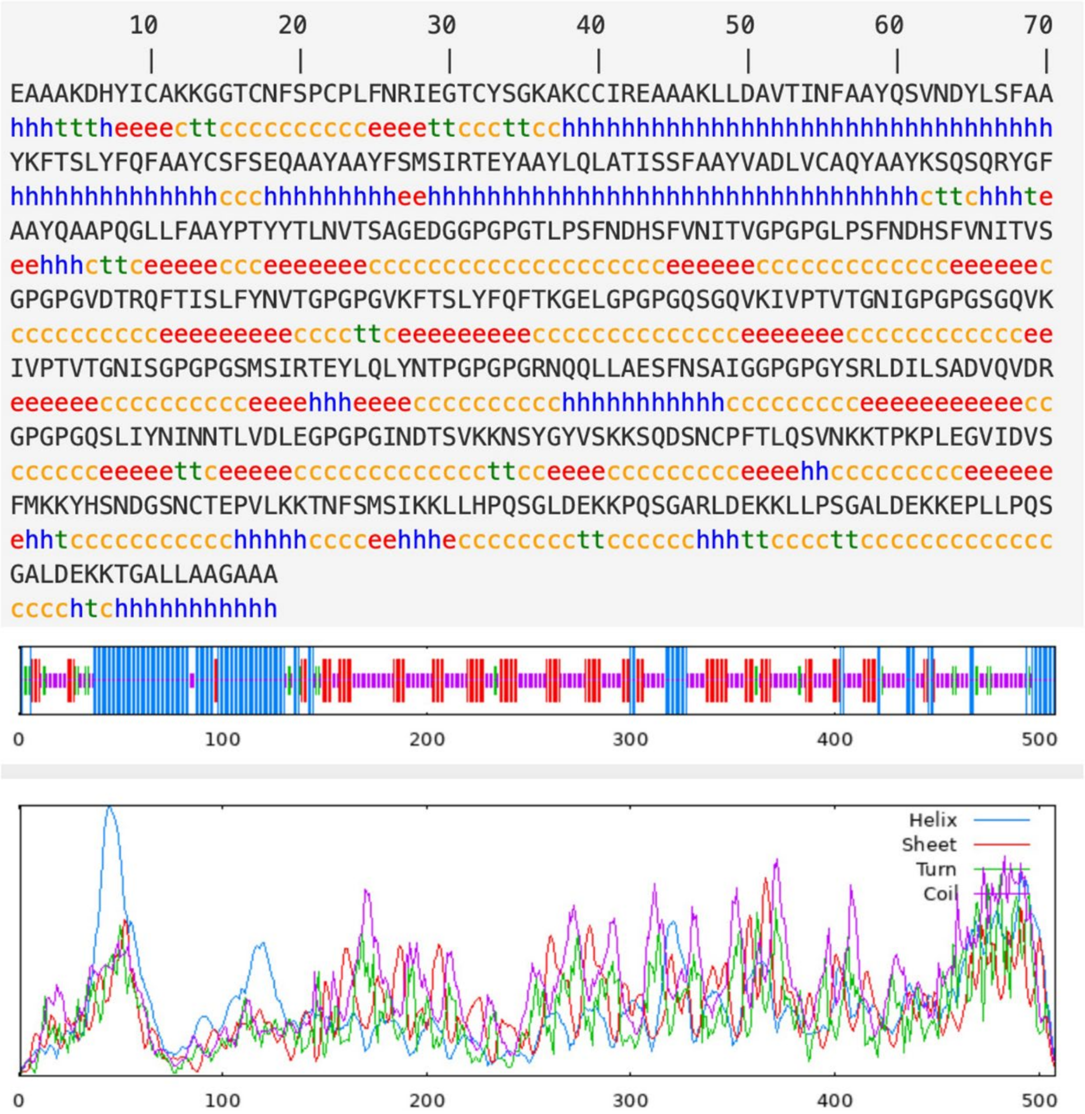

Figure S4. The prediction of *rPMEV* secondary structure. The sequence length of the vaccine construct is 508 amino acids. The blue h is alpha helix and accounts for 27.56%, the red e is extended strand and accounts for 23.03%, the green t is beta turn and accounts for 5.51%, and the yellow c is random coil and accounts for 43.90%.

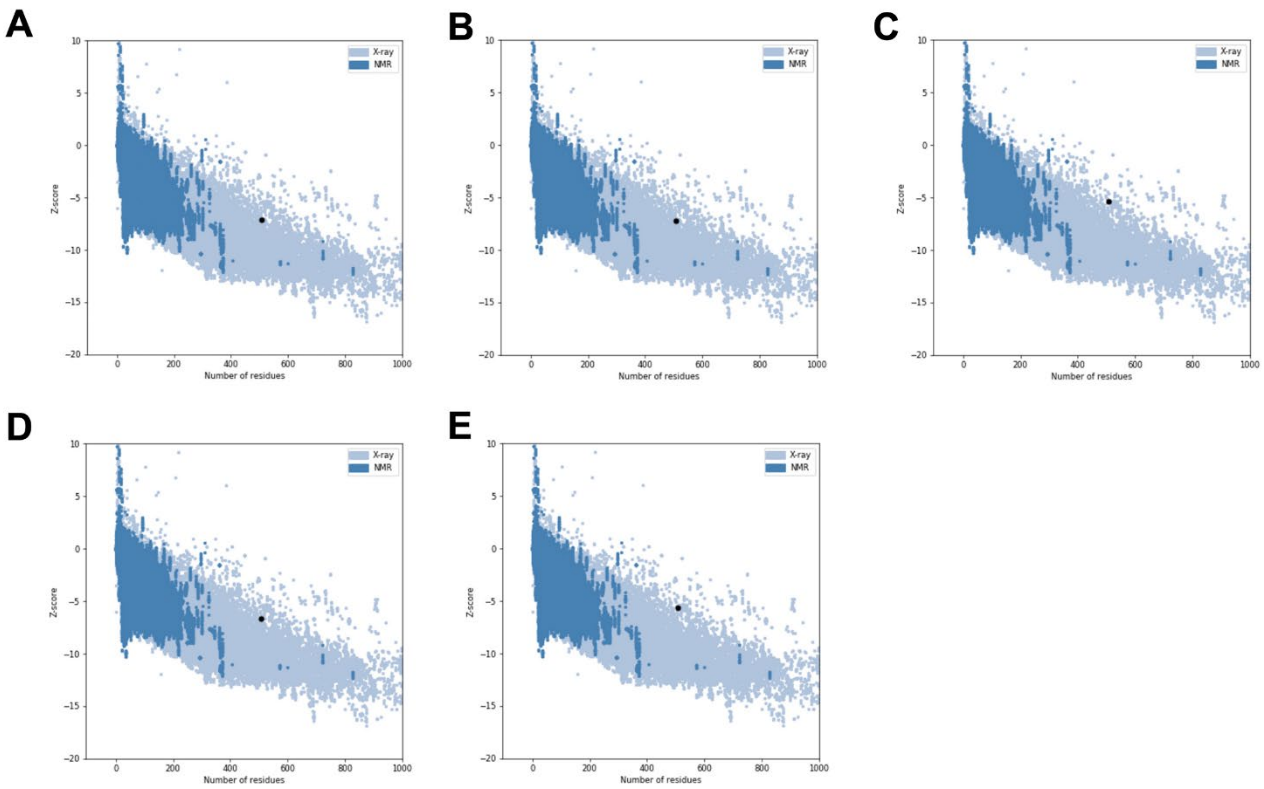

Figure S5. The Z-Score of five models predicted by Robetta software. The Z-Score of Model 1 **(A)** is -7.16, Model 2 **(B)** is -7.2, Model 3 **(C)** is -5.4, Model 4 **(D)** is -6.69, and Model 5 **(E)** is -5.52.

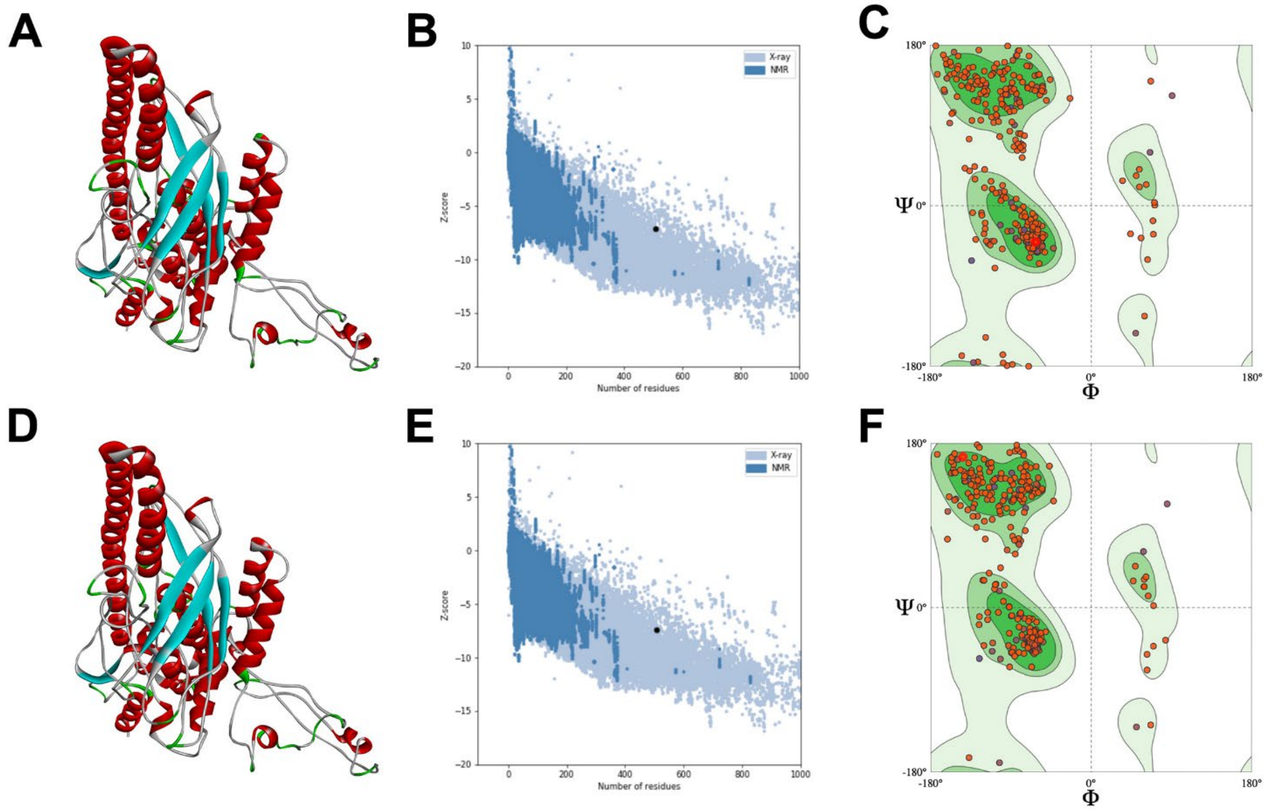

Figure S6. Tertiary structure prediction, refinement, and validation. (A) 3D structure of the initial model prediction by Robetta server. The "red" part is the alpha helix, the "cyan" part is the extended strand, the "green" part is the beta turn and the "gray" part is the random coil in 3D model. **(B)** The Z-Score of the initial model. The Z-Score plot contains Z-Scores of all experimental protein chains in PDB determined by NMR spectroscopy (dark blue) and X-ray crystallography (light blue). **(C)** Ramachandran plots for the initial model. **(D)** 3D structure of the final tertiary structure refined by GalaxyRefine Server. The "red" part is the alpha helix, the "cyan" part is the extended strand, the "green" part is the beta turn and the "gray" part is the random coil in 3D model. **(E)** The Z-Score of the final tertiary structure. The Z-Score plot contains Z-Scores of all experimental protein chains in PDB determined by NMR spectroscopy (dark blue) and X-ray crystallography (light blue). **(F)** Ramachandran plots for final tertiary structure.

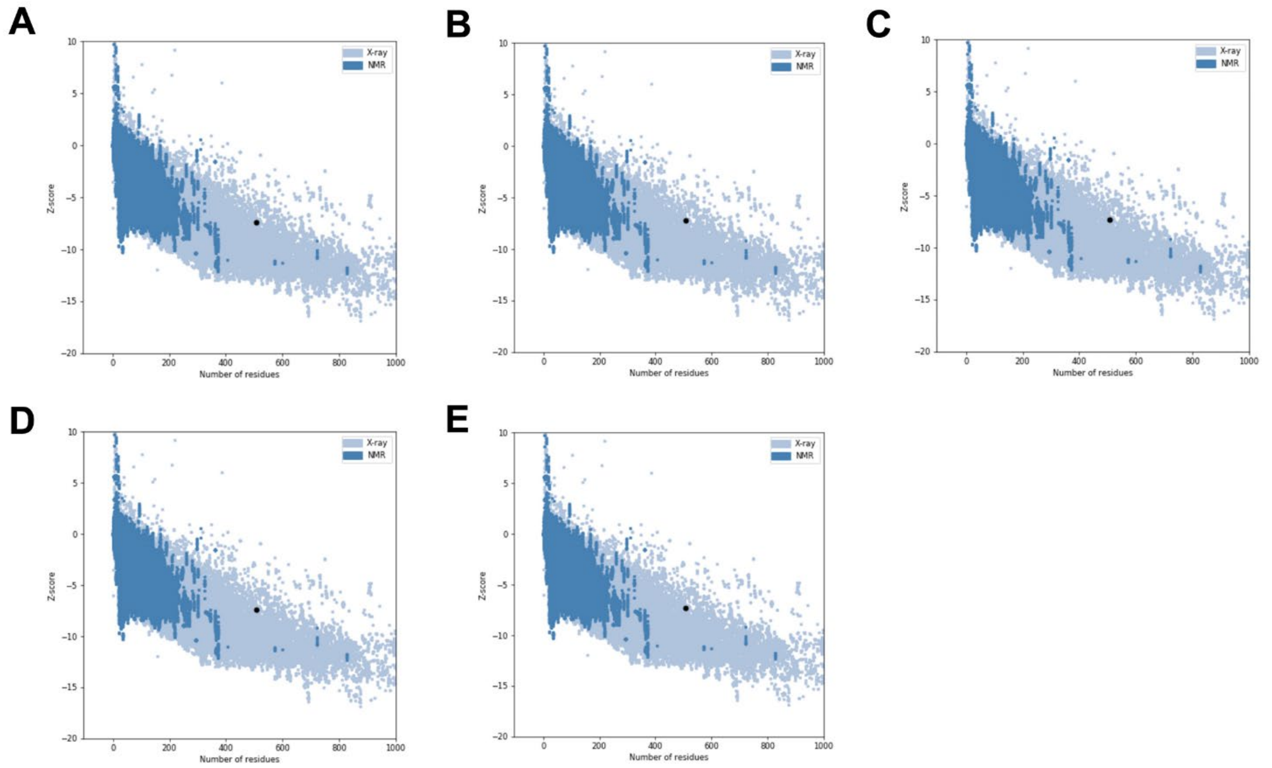

Figure S7. The Z-Score of five optimized models refined by GalaxyRefine Server. The Z-Score of Model 1 **(A)** is -7.39, Model 2 **(B)** is -7.25, Model 3 **(C)** is -7.29, Model 4 **(D)** is -7.36, and Model 5 **(E)** is -7.31.

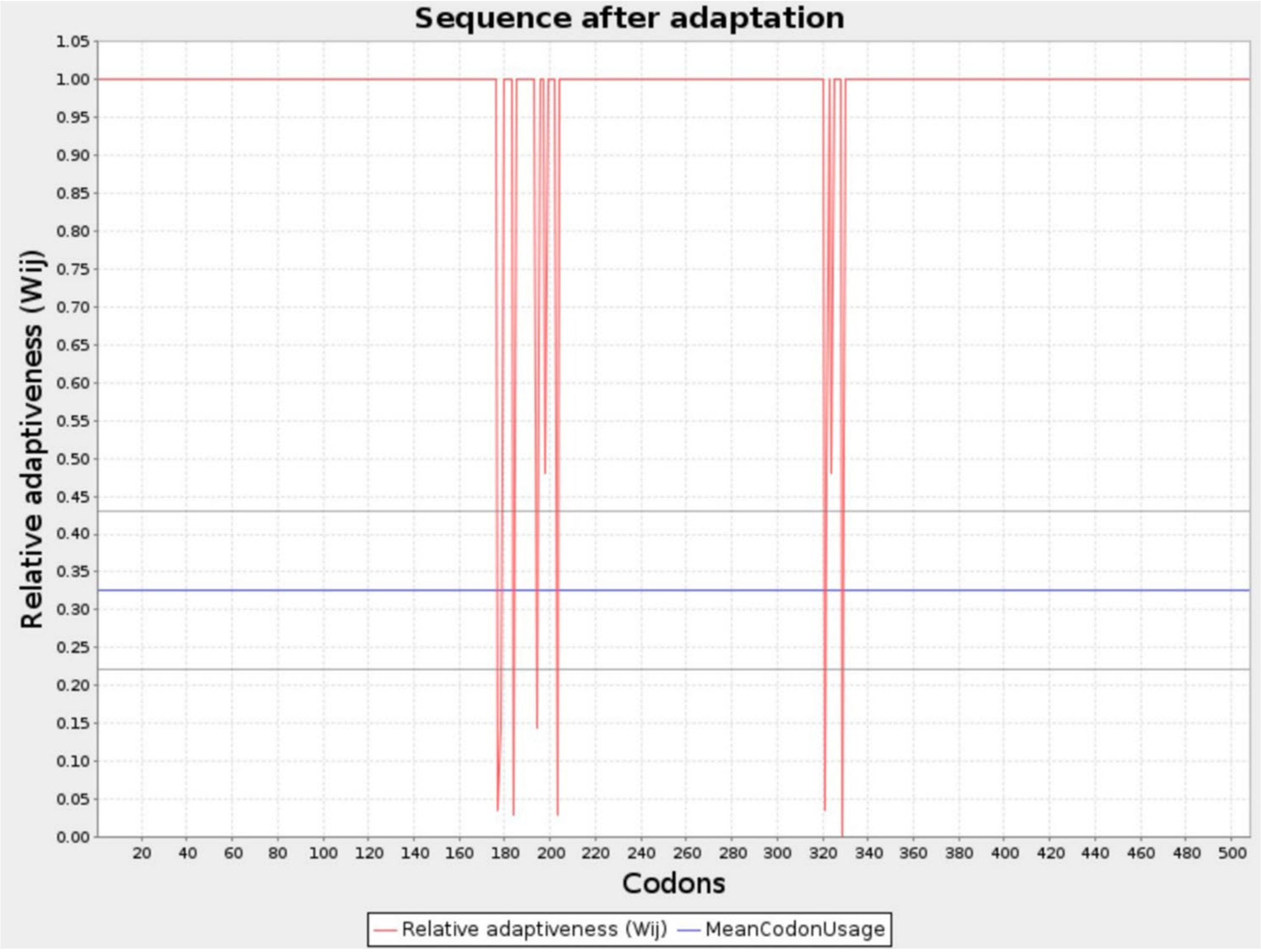

Figure S8. The codon optimization of *rPMEV*.

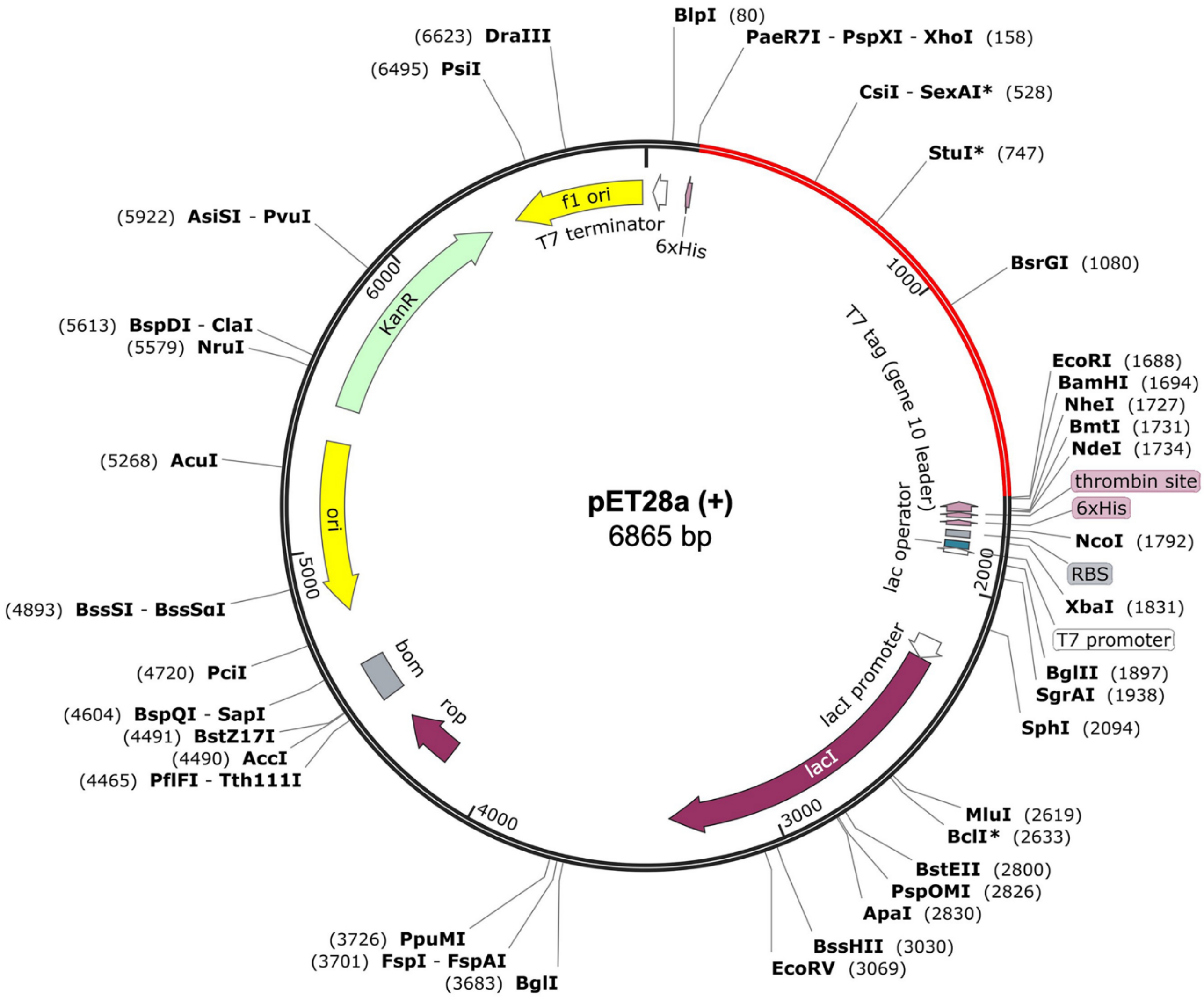

Figure S9. Cloning of designed vaccine into a pET28a (+) vector. The red area represents the PEDV vaccine, while the black area represents the expression vector, pET28a (+).

2.2 Supplementary Tables

Table S1. The antigenic value of PEDV S proteins

PEDV Strain	Genotype	Protein	Accession Number [a]	Residue Length	VaxiJen Score [b]
CH/SCZJ/2018	G2a	S	AZL47228	1386aa	0.4526
LW/L	G2b	S	QBM00061	1383aa	0.4301
CH/SDLQ/09/2020	G2c	S	UDL09544	1385aa	0.4383
CH/SCST/04/2020	G2d	S	UDL09540	1382aa	0.4283

[a] *The accession number from NCBI.*

[b] *VaxiJen v2.0 was used for predicting antigenicity scores with the default threshold of 0.4.*

Table S2. The CTLs of CH/SCZJ/2018

	No.	Position	Sequence	VaxiJen Score [a]
CH/SCZJ/2018	01	400-408	LLDAVTINF	0.9500
	02	559-567	QSVNDYLSF	0.5278
	03	596-604	KFTSLYFQF	1.4349
	04	649-657	NSSFLAGVY	0.4270
	05	682-690	CSFSEQAAY	0.8145
	06	770-778	FSMSIRTEY	1.5744
	07	841-849	LQLATISSF	0.6702
	08	908-916	VADLVCAQY	1.2280
	09	948-956	FTSAAALPF	0.6660
	10	950-958	SAAALPFSY	1.0988
	11	958-966	YAVQARLNY	1.0482
	12	988-996	AIGNITSAF	0.5398
	13	1106-1114	KSQSQRYGF	0.4935
	14	1127-1135	QAAPQGLLF	0.4270

[a] *VaxiJen v2.0 was used for predicting antigenicity scores with the default threshold of 0.4.*

Table S3. The CTLs of LW/L

	No.	Position	Sequence	VaxiJen Score [a]
LW/L	01	398-406	LLDAVTINF	0.9500
	02	546-554	KSQDSNCPF	1.1516
	03	557-565	QSVNDYLSF	0.5278
	04	591-599	SGVKFTSLY	0.6925
	05	594-602	KFTSLYFQF	1.4349
	06	680-688	CSFSEQAAY	0.8145
	07	768-776	FSMSIRTEY	1.5744
	08	839-847	LQLATISSF	0.6702
	09	906-914	VADLVCAQY	1.2280
	10	946-954	FTAAAALPF	0.6857
	11	986-994	AIGNITPAF	1.2310
	12	1104-1112	KSQSQRYGF	0.4935
	13	1125-1133	QAAPQGLLF	0.4270
	14	1173-1181	ELQDTATEY	1.0436
	15	1247-1255	SLDVFNATY	0.5348

[a] *VaxiJen v2.0 was used for predicting antigenicity scores with the default threshold of 0.4.*

Table S4. The CTLs of CH/SDLQ/09/2020

	No.	Position	Sequence	VaxiJen Score [a]
CH/SDLQ/09/2020	01	158-166	FSDKIYHFY	0.5168
	02	321-329	TAFGTNFSF	1.0613
	03	400-408	LLDAVTINF	0.9500
	04	548-556	KSQDSNCPF	1.1516
	05	559-567	QSVNDYLSF	0.5278
	06	593-601	SGVKFTSLY	0.6925
	07	596-604	KFTSLYFQF	1.4349
	08	649-657	NSSFLAGVY	0.4270
	09	682-691	CSFSEQAAY	0.8145
	10	770-778	FSMSIRTEY	1.5744
	11	841-850	LQLATISSF	0.6702
	12	908-916	VADLVCAQY	1.2280
	13	948-956	FTAAAALPF	0.6857
	14	950-958	AAAALPFSY	1.0557
	15	958-966	YAVQARLNY	1.0482
	16	988-994	AIGNITSAF	0.5398
	17	1106-1114	KSQSQRYGF	0.4935
	18	1127-1135	QAAPQGLLF	0.4270

19	1175-1183	ELQDTATEY	1.0436
20	1249-1257	SLDVFNATY	0.5348

[a] *VaxiJen v2.0 was used for predicting antigenicity scores with the default threshold of 0.4.*

Table S5. The CTLs of CH/SCST/04/2020

	No.	Position	Sequence	VaxiJen Score [a]
CH/SCST/04/2020	01	54-62	GVHGIFLSY	0.6922
	02	317-325	TAFGTNLSF	1.0670
	03	396-404	LLDAVTINF	0.9500
	04	544-552	KSQDSNCPF	1.1516
	05	555-563	QSVNDYLSF	0.5278
	06	589-597	SGVKFTSLY	0.6925
	07	592-600	KFTSLYFQF	1.4349
	08	645-653	NSSFLAGVY	0.4270
	09	678-686	CSFSEQAAY	0.8145
	10	766-774	FSMSIRTEY	1.5744
	11	837-845	LQLATISSF	0.6702
	12	904-912	VADLVCAQY	1.2280
	13	944-952	FTAAAALPF	0.6857
	14	946-954	AAAALPFSY	1.0557
	15	954-962	YAVQARLNY	1.0482
	16	984-992	AIGNITSAF	0.5398
	17	1102-1110	KSQSQRYGF	0.4935
	18	1123-1131	QAAPQGLLF	0.4270

[a] *VaxiJen v2.0 was used for predicting antigenicity scores with the default threshold of 0.4.*

Table S6. The HTLs of CH/SCZJ/2018

	No.	Position	Sequence	Vaxijen Score [a]
CH/SCZJ/2018	01	190-204	YEPTYYTLNVTSAGE	0.9492
	02	191-205	EPTYYTLNVTSAGED	0.912
	03	192-206	PTYYTLNVTSAGEDG	0.8008
	04	462-476	LDDGFYPISSRNLLS	0.4504
	05	485-499	TLPSFNDHSFVNITV	0.8490
	06	486-500	LPSFNDHSFVNITVS	0.8535
	07	491-505	DHSFVNITVSASFGG	0.9907
	08	526-540	VDTRQFTISLFYNVT	0.4093
	09	593-607	SVVKFTSLYFQFTKG	0.7784
	10	594-608	VVKFTSLYFQFTKGE	0.9185
	11	595-609	VKFTSLYFQFTKGEL	1.2331
	12	598-612	TSLYFQFTKGELITG	0.6718
	13	599-613	SLYFQFTKGELITGT	0.7398
	14	741-755	SGSIGYVPSQSGQVK	0.9411
	15	742-756	GSIGYVPSQSGQVKI	0.9312
	16	743-757	SIGYVPSQSGQVKIV	0.8473
	17	750-764	QSGQVKIVPTVTGNI	0.7858
	18	751-765	SGQVKIVPTVTGNIS	0.9438
	19	771-785	SMSIRTEYLQLYNTP	0.7639

20	853-867	GYNFTNVLGVSVYDP	0.6562
21	861-875	GVSVYDPAIGRVVQK	0.6475
22	862-876	VSVYDPAIGRVVQKR	0.4669
23	976-990	RNQQLLAESFNSAIG	0.4458
24	1053-1067	YSRLDILSADVQVDR	0.4896
25	1283-1297	QSLIYNINNTLVDLE	0.7696

[a] *VaxiJen v2.0 was used for predicting antigenicity scores with the default threshold of 0.4.*

Table S7. The HTLs of LW/L

	No.	Position	Sequence	Vaxijen Score [a]
LW/L	01	183-197	AMQYVYEPTYYILNV	0.4007
	02	184-198	MQYVYEPTYYILNVT	0.6435
	03	185-199	QYVYEPTYYILNVTS	0.4698
	04	188-202	YEPTYYILNVTSAGE	0.7528
	05	189-203	EPTYYILNVTSAGED	0.7157
	06	190-204	PTYYILNVTSAGEDG	0.5961
	07	483-497	TLPSFNDHSFVNITV	0.8490
	08	484-498	LPSFNDHSFVNITVS	0.8535
	09	489-503	DHSFVNITVSAAFGG	0.9377
	10	524-538	VDTRQFTISLFYNVT	0.4093
	11	590-604	GSGVKFTSLYFQFTK	0.9828
	12	591-605	SGVKFTSLYFQFTKG	0.9822
	13	592-606	GVKFTSLYFQFTKGE	1.2002
	14	593-607	VKFTSLYFQFTKGEL	1.2331
	15	596-610	TSLYFQFTKGELITG	0.6718
	16	697-611	SLYFQFTKGELITGT	0.7398
	17	740-754	GSIGYVRSQSGQVKI	0.7544
	18	741-755	SIGYVRSQSGQVKIA	0.7121
	19	748-762	QSGQVKIAPTVTGNI	0.9223

20	749-763	SGQVKIAPTVTGNIS	1.0804
21	769-783	SMSIRTEYLQLYNTP	0.7639
22	851-865	GYNFTNVLGVSVYDP	0.6562
23	859-873	GVSVYDPARGRVVQK	0.4858
24	943-957	LGGFTAAAALPFSHA	0.5721
25	947-988	RNQQLLAESFNSAIG	0.4458
26	1051-1065	YSRLDILSADVQVDR	0.4896
27	1246-1260	LSLDVFNATYFNLTG	0.7977
28	1247-1261	SLDVFNATYFNLTGE	0.7833
29	1248-1262	LDVFNATYFNLTGEI	0.6602
30	1249-1263	DVFNATYFNLTGEIA	0.7002
31	1280-1294	QSLIYNINNTLVDLE	0.7696

[a] *VaxiJen v2.0 was used for predicting antigenicity scores with the default threshold of 0.4.*

Table S8. The HTLs of CH/SDLQ/09/2020

	No.	Position	Sequence	Vaxijen Score [a]
CH/SDLQ/09/2020	01	190-204	YEPTYYMLNVTSAGE	0.84
	02	191-205	EPTYYMLNVTSAGED	0.8028
	03	192-206	PTYYMLNVTSAGEDG	0.6812
	04	462-476	LDDGFYPISSRNLLS	0.4504
	05	485-499	TLPSFNDHSFVNITV	0.849
	06	486-500	LPSFNDHSFVNITVS	0.8535
	07	491-505	DHSFVNITVSASFGG	0.9907
	08	526-540	VDTRQFTISLFYNVT	0.4093
	09	592-606	GSGVKFTSLYFQFTK	0.9828
	10	593-607	SGVKFTSLYFQFTKG	0.9822
	11	594-608	GVKFTSLYFQFTKGE	1.2002
	12	595-609	VKFTSLYFQFTKGEL	1.2331
	13	598-612	TSLYFQFTKGELITG	0.6718
	14	599-613	SLYFQFTKGELITGT	0.7398
	15	743-757	SIGYVSSQSGQVKIA	0.9704
	16	750-764	QSGQVKIAPTVTGNI	0.9223
	17	751-765	SGQVKIAPTVTGNIS	1.0804
	18	771-785	SMSIRTEYLQLYNTP	0.7639
	19	861-875	GVSEYDPASGRVVQK	0.8743

20	862-876	VSEYDPASGRVVQKR	0.6967
21	976-990	RNQQLLAESFNSAIG	0.4458
22	1053-1067	YSRLDILSADVQVDR	0.4896
23	1282-1296	QSLIYNINNTLVDLE	0.7696

[a] *VaxiJen v2.0 was used for predicting antigenicity scores with the default threshold of 0.4.*

Table S9. The HTLs of CH/SCST/04/2020

	No.	Position	Sequence	Vaxijen Score [a]
CH/SCST/04/2020	01	186-200	YTPTYYMLNVTSAGE	0.6888
	02	187-201	TPTYYMLNVTSAGED	0.6719
	03	188-202	PTYYMLNVTSAGEDG	0.6812
	04	458-472	LDDGFYPISSRNLLS	0.4504
	05	481-495	TLPSFNDHSFVNITV	0.849
	06	482-496	LPSFNDHSFVNITVS	0.8535
	07	487-501	DHSFVNITVSASFGG	0.9907
	08	522-536	VDTRQFTISLFYNVT	0.4093
	09	588-602	GSGVKFTSLYFQFTK	0.9828
	10	589-603	SGVKFTSLYFQFTKG	0.9822
	11	590-604	GVKFTSLYFQFTKGE	1.2002
	12	591-605	VKFTSLYFQFTKGEL	1.2331
	13	595-609	SLYFQFTKGELITST	0.6927
	14	737-751	SGSIGYVPSQSGQVK	0.9411
	15	738-752	GSIGYVPSQSGQVKI	0.9312
	16	739-753	SIGYVPSQSGQVKIA	0.8889
	17	746-760	QSGQVKIAPTVTGNI	0.9223
	18	747-761	SGQVKIAPTVTGNIS	1.0804
	19	767-781	SMSIRTEYLQLYNTP	0.7639

20	849-863	GYNFTNVLGVSVYDP	0.6562
21	857-871	GVSVYDPASGRVVQK	0.605
22	858-872	VSVYDPASGRVVQKR	0.4243
23	972-986	RNQQMLAESFNSAIG	0.4681
24	1049-1063	YSRLDILSADVQVDR	0.4896
25	1279-1293	QSLIYNINNTLVDLE	0.7696

[a] *VaxiJen v2.0 was used for predicting antigenicity scores with the default threshold of 0.4.*

Table S10. The LBEs of vaccine construction

No.	Sequence	VaxiJen Score [a]
01	INDTSV	1.1931
02	NSYGYVS	0.4527
03	SQDSNCPFTLQSVN	1.2968
04	TPKPLEGVIDVSFM	0.6367
05	YHSNDGSNCTEPVL	0.5062
06	TNFSMSI	1.4345

[a] *VaxiJen v2.0 was used for predicting antigenicity scores with the default threshold of 0.4.*

Table S11. The CBEs of vaccine construction

No.	Peptide Sequence	PEDV Strain
01	L474, L475, H477, P714, Q750, S751, G752, L1234, D1235, E1236	CH/SCZJ/2018
02	P712, Q748, S749, G750, A866, R867, L1231, D1232, L1231	LW/L
03	L474, L475, P714, S751, G752, A868, L1233, D1234, E1235	CH/SDLO/09/2020
04	E76, P77, L470, L471, P710, Q746, S747, G748, A864, L1230, D1231, E1232	CH/SCST/04/2020

Table S12. The result of molecular docking between *rPMEV* and TLR4

Cluster	Members	Representative	Weighted Score
0	42	Lowest Energy	-1046.1
1	39	Lowest Energy	-1015.5
2	36	Lowest Energy	-996.5
3	35	Lowest Energy	-939.9
4	34	Lowest Energy	-995.9
5	32	Lowest Energy	-871.6
6	29	Lowest Energy	-933.5
7	25	Lowest Energy	-985.7
8	24	Lowest Energy	-875
9	21	Lowest Energy	-892
10	20	Lowest Energy	-868.3
11	20	Lowest Energy	-956.7
12	20	Lowest Energy	-879.2
13	19	Lowest Energy	-882.2
14	19	Lowest Energy	-843.8
15	18	Lowest Energy	-876.5
16	15	Lowest Energy	-835.8
17	15	Lowest Energy	-985.8
18	14	Lowest Energy	-869.3

19	13	Lowest Energy	-865.9
20	13	Lowest Energy	-821.4
21	12	Lowest Energy	-845.9
22	12	Lowest Energy	-836
23	12	Lowest Energy	-816.5
24	11	Lowest Energy	-818.5
25	11	Lowest Energy	-836.1
26	10	Lowest Energy	-880.3
27	10	Lowest Energy	-823
28	9	Lowest Energy	-822.9
29	8	Lowest Energy	-797.7
